# Comparison of Five Major Trichome Regulatory Genes in *Brassica villosa* with Orthologues within the *Brassicaceae*

**DOI:** 10.1371/journal.pone.0095877

**Published:** 2014-04-22

**Authors:** Naghabushana K. Nayidu, Sateesh Kagale, Ali Taheri, Thushan S. Withana-Gamage, Isobel A. P. Parkin, Andrew G. Sharpe, Margaret Y. Gruber

**Affiliations:** 1 Agriculture and Agri-Food Canada, Saskatoon Research Centre, Saskatoon, SK, Canada; 2 Department of Biology, University of Saskatchewan, Saskatoon SK, Canada; 3 National Research Council (NRC), Saskatoon SK, Canada; 4 POS Bio-Sciences, Saskatoon, SK, Canada; University of Michigan, United States of America

## Abstract

Coding sequences for major trichome regulatory genes, including the positive regulators *GLABRA 1*(*GL1*), *GLABRA 2* (*GL2*), *ENHANCER OF GLABRA 3* (*EGL3*), and *TRANSPARENT TESTA GLABRA* 1 (*TTG1*) and the negative regulator *TRIPTYCHON* (*TRY*), were cloned from wild *Brassica villosa*, which is characterized by dense trichome coverage over most of the plant. Transcript (FPKM) levels from RNA sequencing indicated much higher expression of the *GL2* and *TTG1* regulatory genes in *B. villosa* leaves compared with expression levels of *GL1* and *EGL3* genes in either *B. villosa* or the reference genome species, glabrous *B. oleracea*; however, cotyledon *TTG1* expression was high in both species. RNA sequencing and Q-PCR also revealed an unusual expression pattern for the negative regulators *TRY* and CPC, which were much more highly expressed in trichome-rich *B. villosa* leaves than in glabrous *B. oleracea* leaves and in glabrous cotyledons from both species. The *B. villosa* TRY expression pattern also contrasted with TRY expression patterns in two diploid Brassica species, and with the Arabidopsis model for expression of negative regulators of trichome development. Further unique sequence polymorphisms, protein characteristics, and gene evolution studies highlighted specific amino acids in *GL1* and *GL2* coding sequences that distinguished glabrous species from hairy species and several variants that were specific for each *B. villosa* gene. Positive selection was observed for GL1 between hairy and non-hairy plants, and as expected the origin of the four expressed positive trichome regulatory genes in *B. villosa* was predicted to be from *B. oleracea*. In particular the unpredicted expression patterns for *TRY* and *CPC* in *B. villosa* suggest additional characterization is needed to determine the function of the expanded families of trichome regulatory genes in more complex polyploid species within the *Brassicaceae*.

## Introduction

Trichomes are protruding structures which protect plant surfaces against dehydration and insect pests, and provide a storage organ to handle metal toxicity [1], [2]. Trichome development has been studied extensively at the molecular level in non-glandular trichomes of the model plant, *Arabidopsis thaliana*, in which trichome regulation is controlled by multiple transcription factors. Among these, a number of major positive regulatory genes for trichome initiation have been studied in detail, including *GLABRA 1*(*GL1*), *GLABRA 2* (*GL2*), *GLABRA 3* (*GL3*), *ENHANCER OF GLABRA 3* (*EGL3*), and *TRANSPARENT TESTA GLABRA* 1 (*TTG1*) [3]. *GL1* was isolated by gene tagging [4] and is a member of the R2R3 activator MYB gene family in *A. thaliana* [5]. *GL3* and *EGL3* encode members of an IIIf subfamily of basic Helix-Loop-Helix (bHLH) proteins [6]. *TTG1* encodes a WD40 domain protein [7]. GL1, TTG1 and GL3/EGL3 are positive patterning proteins which form an activator MYB-bHLH-WD40 (MBW) tri-protein complex that induces the expression of an immediate downstream target gene, *GLABRA 2* (*GL2*). *GL2* encodes a homeobox transcription factor and its induction is required both for trichome cell specification and for subsequent phases of trichome morphogenesis such as cell expansion, branching, and cell wall maturation [8]–[10]. Mutations in any of these four above-mentioned genes reduce trichome initiation and the density of trichome patterning in *A. thaliana* [11]–[14] A yeast two hybrid assay demonstrated a direct physical interaction between *GL3* and *GL1*, *TTG1*, and itself; *GL1* and *GL3* co-overexpression confirmed their interaction, but *GL1* and *TTG1* do not physically interact [15]. Mutations in *GL3* modestly reduce trichome density, branching, DNA endoduplication, and trichoblast size [11], [15]. *Egl3* mutant plants have no obvious trichome defects, but *gl3egl3* double mutants show a complete glabrous phenotype [14] due to some functional overlap between the two genes [16].

*TRIPTYCHON* (*TRY*) is the first identified negative regulator of Arabidopsis trichome initiation [11] and encodes a small single R3-MYB repeat protein which lacks the R2 activation domain [6], [17]. The activator proteins of the Arabidopsis MBW tri-protein complex are assumed to locally activate their own expression and that of *TRY* [18]. TRY moves through plasmodesmata into adjacent cells where it and several other potent inhibitor proteins such as CAPRICE (CPC), ENHANCER OF TRY and CPC1 and 2 (ETC1 and ETC3) can competitively bind to the MBW complex to release GL1 [19]–[23]. This release of GL1 renders the MYB complex inactive at stimulating GL2 expression and prevents trichome initiation in neighbouring cells [2], [18]. At the same time, the GL3 protein “traps” and reduces the mobility of TTG1 proteins (which can move from neighboring cells into trichome-initiating cells) by binding to them in trichome-initiating cells [3], [24], [25].

Trichomes are found on other species within the *Brassicaceae*, but very few details are known about *Brassica* trichome genes and their regulation compared with the model Arabidopsis. *Brassica napus* is an amphidiploid species originating from a cross between *Brassica rapa* (of the A genome) and *Brassica oleracea* (of the C genome) [26]. Leaf trichomes are found in substantial numbers on many lines of *B. rapa*, but not on *B. oleracea*, and the distribution on *B. rapa* is patchy rather than even, the patterning is light, and occurs mainly on only a few tissues (eg. leaves and stems) [27]. Leaf trichomes are mainly found on the adaxial side of the *A. thaliana* and *B. rapa* leaves. In contrast, *B. napus* is practically devoid of trichomes.

Trichome genes that have been characterized from the *Brassicas* only include *B. rapa TTG1*, *GL1*, and *GL2*, but analysis of a full set of regulatory genes has never been conducted within a single species. An orthologue, *BrTTG1*, isolated from a brown-seeded hairy *B. rapa* genotype, was found to functionally complement (rescue) an *A. thaliana ttg1* mutant [28], while the orthologue isolated from *B. rapa* yellow-seeded glabrous germplasm was not functional. *B. rapa* leaf hairiness was associated with nucleotide polymorphisms in the DNA-binding domain of the *BrGL1* gene [29], and several *BrGL1* alleles with varying impact on phenotype have been found. *B. rapa* harbouring the B-allele of *BrGL1* produces hairy plants when transformed with the A-allele and hairless plants with the C-allele [30]. *GL2* promoter regions in *A. thaliana* and *B. napus* have low homology and confer differential expression patterns, such that the *BnGL2* promoter introduced into *A. thaliana* is only expressed at an early stage of trichome development, whereas the native *AtGL2* promoter is expressed throughout the whole process of trichome development [31]. Moreover, glabrous *B. napus* plants expressing the *A. thaliana GL3* from a constitutive promoter develop an extremely dense coverage of trichomes on stems and young leaves [32] compared with overexpression in Arabidopsis. These findings indicate differences between the *Brassica* and *A. thaliana* genes that can impact trichome function and trichome patterning.

*Brassica villosa* is a wild C genome relative of glabrous *B. oleracea*, yet *B. villosa* is even more densely covered, with evenly distributed trichomes over most surfaces of the plant, than *B. napus* transformed with the *AtGL3* gene [32], [33]. Like other *Brassica* species, *B. villosa* trichomes are without branches, whereas trichomes on the model species, *A. thaliana*, are multi-branched. The limited information available for *Brassica* trichome genes and the dense trichome patterning on *B. villosa* organs prompted us to dissect the trichome regulatory gene network in this species. In this regard, we successfully amplified *B. villosa* orthologues to the *GL1*, *GL2, EGL3, TTG1* and *TRY* coding sequences and compared their expression patterns and sequence structure with corresponding genes available from *B. rapa*, *B. oleracea*, *B. napus*, and *A. thaliana*.

## Materials and Methods

### Plant Material

*A. thaliana* (Columbia), *Brassica oleracea* (T01000 DH3), and *Brassica napus* (cv. Westar) seeds were obtained from germplasm collections at the Saskatoon Research Centre, Saskatoon, Canada. *Brassica villosa* Biv. subsp. *drepanensis* seeds were obtained from the Centre for Genetic Resources, Wageningen, The Netherlands. Seeds of *B. rapa* (cv. Echo) were obtained from Plant Gene Resources Canada, Saskatoon, Canada. All seeds were grown in soil-less potting mixture in a controlled greenhouse environment (16 h light/8 h dark, 20/17°C) supplemented with halogen lights at Agriculture and Agri-Food Canada, Saskatoon, Canada.

### Amplification and Sequence Analysis of Five Major Trichome Genes from B. villosa

Specific primers to amplify *Brassica villosa* (*Bv*) orthologues to *GL1*, *GL2*, *EGL3, TTG1* and *TRY* were designed from coding sequences from *B. napus* and *B. rapa* (Table 1). The orthologues were amplified from cDNA developed from *B. villosa* seedling leaf RNA (isolated as described under gene expression profiling). At the time of amplification, the *B. rapa EGL3* (accession HM208589.1) was mis-annotated as *GL3* in NCBI, but the nomenclature was recently corrected. PCR was conducted using 32 cycles of 94°C for 30 sec, 56°C for 30 sec, and 72°C for up to 3 minutes (based on the length of the template cDNA), a final extension time of 5 minutes at 72°C, and PFU Ultra II fusion HotStart DNA polymerase enzyme (Stratagene, USA). Primers were designed starting at the translational start and ending with the 3′ ends of the *B. napus* and *B. rapa* cDNA sequences (including restriction sites) except for *TRY*, for which the forward primer was designed from the 72^nd^ residue upstream from the translational start site (Table 1). At the time of cloning, only an unannotated EST was available for *TRY* rather than a cDNA with a verified translational start site. Amplified sequences were cloned into the pGEM-Teasy vector (Promega, Madison, WI, USA), and then several amplicons (clones) per gene were sequenced in the DNA Technologies Laboratory of National Research Council, Saskatoon, and used to search the NCBI database using BLASTN to determine gene identity.

**Table 1:** Primers used to isolate/analyze *GL1*, *GL2*, *GL3*, *TTG1* and *TRY* genes from *B. villosa*.

Primers	NCBI accession	Sequence (5′ to 3′)
*Coding Sequences from B. napus* 1		
GL1-F	HQ162473.1	GTCAGGATCCATGAGAACGAGGAGAAGAACAGA
GL1-R		GTCACTGCAGCTAGAGACAGTAGCCAGTATCA
GL2-F	EU826520.1	GTCAGGATCCATGTCAATGGCCGTCGAGATGTCA
GL2-R		ATATCTGCAGTGTTGTGCAGCGTGACAGAGACGA
TRY-F	EE451172.1	AGCTGGATCCGCTTGCATTCTCCAACT
TRY-R		CGCACTGCAGGCAATTTCGTTATGCTATATG
*Coding Sequences from B. rapa* 1		
EGL3-F	HM208589.1	GTCAGGATCCATGGCTGCTGTAGAAAACAG
EGL3-R		GCAGCTGCAGAGTGCATCTTGAATCATTCCT
TTG1-F	HM208590.1	TAGAGGATCCATGGACAACGCAGCTCCGGACT
TTG1-R		AGTCGGTACCTCAAACTCTAAGGAGCTGCA
*Conserved Regions (for Q-PCR)* 2		
GL1-F		ACTGGGCTGAAGAGGTGTGGA
GL1-R		GAGATGAGTGTTCCAGTGA
GL2-F		CGCTGGCCGGGAGAAAGAGC
GL2-R		GGAGGTTTTTTCTGGATGAA
EGL3-F		ACATTCAATGGAGTTACGGA
EGL3-R		AGAGATTCGTAAAGCTCTCT
TTG1-F		CTCTGGGAGGTCAACGAA
TTG1-R		ATGCTGCACGTGCCTAAC
TRY-F		CATCACTCCTCTTCTCACA
TRY-R		TGTGGTGGGGAAGAAAACAGA
BnEF1F (*B. napus*)		CCCATTCGTCCCCATCTCTGGA
BnEF1R (*B. napus*)		ACGGAGGGGCTTGTCCGAGG

1 Forward (F) primers were designed at the 5′ coding end and reverse (R) primers to the 3′ end of cDNA sequences from *B. napus* for GL1 and GL2 and from *B. rapa* for EGL3 and TTG1. Forward primers for TRY were designed 72 nucleotides before coding region and reverse primer at 3′ end of the cDNA from *B. rapa.* Underlined sequences indicate incorporated restriction enzyme sites; *Bam*HI and *Pst*I sites in the GL1, GL2, EGL3 and TRY forward and reverse primers, respectively; *BamH*I and *Kpn*I sites in the TTG1 forward and reverse primers, respectively.

2 Primers for Q-PCR were designed to conserved regions based on alignments between the *A. thaliana* homologue and the homologues from four *Brassica* species. B nEF1F/BnEF1R are endogenous reference gene primers.

### Construction of Transgenic Expression Vectors and Transformation into the Arabidopsis Wild Type

Binary vectors pMP79-103 and pBI121 were initially modified by inserting a 1.7 kb Hydroperoxide Lyase (HPL) [34] promoter between *Hind*III/*Xba*I sites, and then a 2.2 kb *BvGL2* and 506 bp *BvTRY* cDNA sequence were cloned, respectively, downstream of the promoter using *BamH*I/*Kpn*I restriction sites. In a separate construct, pMP79-103 vector was modified by inserting a 2.5 kb Elongation factor 1(EF1) promoter between *Hind*III/*Xba*I sites, and then a 1.8 kb *BvEGL3* cDNA sequence was cloned using *BamH*I/*Pst*I restriction sites. pCAMBIA1305.2 vector was modified by cloning a cassette carrying 570 bp NOS promoter, 1.1 kb cDNA clone of *TTG1* and 400 bp NOS terminator between *EcoR*I and *Hind*III restriction sites. All of these binary constructs were individually transformed into *Agrobacterium tumefaciens* strain GV3101 by electroporation. Arabidopsis plants were transformed with respective plasmids using the floral dip method [34]. Transformed plants were selected on the basis of respective vector based resistance markers. Pictures were taken from T1 generation plants three weeks after germination.

### RNA Sequencing for Verification and Expression Profiling of Individual Gene Copies

Fresh-frozen cotyledons and first true-leaves (before the second true leaf emergence) of *B. villosa* and *B. oleracea* were collected for RNA sequencing from seedlings (5–7 plants per replicate) for 3 biological replicates. RNA was extracted using standard methods, then developed into libraries using a TruSeq RNA sample preparation kit (Illumina, San Diego, CA, USA). Library sequencing (100 cycles) was conducted from both ends on an Illumina HiSeq 2000. A total of 51 Gb of *B.oleracea* reads (Cotyledons; 26 Gb and true leaves; 25 Gb) and 60 Gb of *B.villosa* reads (Cotyledons; 33 Gb and true leaves; 27 Gb) were obtained. The RNAseq data was trimmed using trimmomatic ver.0.30 with minimum quality score of 15, removing the first 12 bp, and a minimum length of 20, which was then aligned to the genome of *B. oleracea* with TopHat ver. 2.0.7 (with parameters, including minimum intron length (i) of 20, maximum intron length (I) of 11000, and distance between pair ends (r) of 30) [35]. The aligned RNAseq reads were assembled into transcripts and their relative abundance was estimated as fragments per kilobase of exon per million fragments mapped (FPKM) using Cufflinks software [35]. Differential expression and statistical analysis of individual gene copies was conducted using Cummerbund package in R; http://www.r-project.org/. Raw data from the RNAseq experiment was deposited to NCBI with SRA accession number, SRP035213.

### Q-PCR for Total Gene Expression Profiling Relative to B. napus

First true leaves were collected separately from three individual plants at the same stage as mentioned above for *B. villosa* for each of the four other species mentioned under plant material. Total RNA was extracted from the seedling leaves (three independent preparations per species) with a commercial RNA-Easy mini kit (Qiagen, Valencia, CA, USA) and cDNA synthesized using Superscript II™ (Invitrogen, Carlsbad, CA) according to manufacturer’s instructions. Quantitative real time-PCR (Q-PCR) was conducted using a Platinum SYBR Green Super Mix-UDG kit (Invitrogen), a CFX96 Real-Time PCR system (BioRad, Hercules, CA, USA), and primers to conserved regions for *GL1, GL2, EGL3, TTG1* and *TRY* within all five species (Table 1). Even though the *B. napus EGL3* coding sequence was not available, the primers to *EGL3*-specific conserved regions in other species amplified the *B. napus* transcripts in Q-PCR reactions. For each pair of gene-specific primers, melting curve analysis was conducted to determine the melting temperature and to ensure a single PCR amplicon of the expected length. Three independent RNA preparations were assayed per species and data was analyzed using CFX Manager Software (BioRad). The expression level of each mRNA was determined using the mean cycle threshold (ΔCT) value normalized to an endogenous reference gene, *BnEF1* (Table 1). Mean values with corresponding standard errors were expressed relative to glabrous *B. napus* leaf tissue and analysed by one-way analysis of variance (ANOVA) using PROC GLM in SAS ver. 9.2 (SAS, 2008) and a completely randomized design (CRD) (with genes and species as the two main factors). Significantly different means were detected by Fisher’s protected Least Significant Difference (LSD) tests.

### Bioinformatic Analysis of Brassica Trichome Regulatory Genes

*A. thaliana* nucleotide and protein sequence information for GL1, GL2, EGL3, TTG1, and TRY was collected from http://www.arabidopsis.org. *B. villosa* sequences were obtained as above. *B. napus* sequences for GL1, GL2, TTG-2 and TRY were obtained from EST databases available at http://rapa.agr.gc.ca and http://blast.ncbi.nlm.nih.gov. *B. napus* EST databases were searched at http://napus.agr.gc.ca/aped (*B. napus EGL3* sequence was unavailable). *B. rapa* sequence information was collected from http://brassicadb.org/brad, http://www.plantgdb.org/BrGDB, and http://www.brassica-rapa.org/BRGP/index.jsp. *B. oleracea* sequences were collected from the *B. oleracea* genome database at Agriculture and Agri-Food Canada, Saskatoon (Parkin, unpublished). Nucleotide and translated protein sequences were aligned using Clustal-W in Vector NTI 9 (Invitrogen). Molecular weight (M*r*) and isoelectric point [35] were determined on translated protein sequences using http://web.expasy.org/compute_pi/. Amino acid frequencies were determined using http://emboss.bioinformatics.nl/cgi-bin/emboss/fuzzpro. Phylogenetic analysis was carried out using Molecular Evolutionary Genetics Analysis software (MEGA v5.1) and the neighbour joining method of phylogenetic inference with a bootstrap parameter of 1000 replications [36]. Initially, all six copies (ESTs) for *BnTTG1* and the short (<100 bp) *B. rapa* sequences for *BrTRY2* and *BrTRY3* were used in phylogeny testing, but later only the longer overlapping *BnTTG1* consensus sequence and the longer *BrTRY1* were used with the other orthologues to improve the confidence (bootstrap values) of phylogenetic relationships above 50%. Protein conserved domain recognition was determined at http://www.ebi.ac.uk/Tools/pfa/iprscan/.

Evolutionary analysis was performed to obtain more information on similarity and variation between the five major trichome regulatory genes of *B. villosa* and orthologues within three other *Brassicas* and *A. thaliana*. A pairwise comparison of *GL1*, *GL2*, *EGL3*, *TTG1* and *TRY* coding region of the orthologous genes was used to calculate the ratio of non-synonymous amino acid substitution rate (*Ka*) to synonymous substitution rate (*Ks*) using the maximum likelihood algorithm implemented in Phylogenetic Analysis by Maximum Likelihood (PAML) [37]. As with the phylogenetic analysis, all the *B. napus TTG1* coding sequences and the very short *B. rapa TRY2* and *TRY3* sequences were initially aligned with other sequences, but later excluded from the evolutionary analysis due to lack of sufficient overlapping sequence. Generally, a *Ka*/*Ks* of 1 indicates neutral selection, *Ka*/*Ks* <1 indicates a functional constraint with purifying selection, and *Ka*/*Ks* >1 shows accelerated evolution with positive selection [37].

## Results

### Trichome Regulatory Gene Amplification in B. villosa

Amplification of the coding regions for *B. villosa* orthologues to the *GL1*, *GL2*, *EGL3*, *TTG1* and *TRY* regulatory genes using primers based on available *B. napus* and *B. rapa* sequences gave single PCR bands (in some cases amplifying multiple copies). The bands were cloned, sequenced and used to search the NCBI database to confirm gene identity and to determine copy number. Three independent clones each were sequenced for the *BvGL1*, *BvGL2*, and *BvTTG1* genes from *B. villosa*, and this was expanded to 10 clones for *BvEGL3*, and thirty clones for *BvTRY*. Alignments of the cloned sequences to private (*B. oleracea*) and public (*B. rapa*, *B. napus*) databases, plus RNAseq data, indicated that *B. villosa* contained a single copy each for *GL1* and *GL2* and two unique sequences for *EGL3* (95% amino acid identity) (Table 2; Figure S1, protein sequence shown). Only one *TTG1* copy could be isolated from *B. villosa* using the *B. rapa* primers in spite of a second copy (with only 45% nucleotide identity) in the *B. oleracea* database (Parkin, unpublished). Sequencing of 30 *TRY* clones indicated two copies from *B. villosa* with 100% amino acid identity (Figure S1). RNAseq suggested a 3rd copy for *B. villosa TRY* not seen within the 30 amplicons (Table 2).

**Table 2:** Database accession numbers for orthologues to the major trichome regulatory genes (*GL1*, *Gl2*, *EGL3*, *TTG1* and *TRY*) in *B. villosa.*

Gene	*B. villosa* (NCBI)	*B. oleracea* (Unpublished data, Dr. I. Parkin)	*B. rapa* (BRAD)	*B. napus* (NCBI)	Arabidopsis(TAIR)	Function in Overexpression And Knockdown studies in *B. rapa*
						
GL1	KF188209	Bo7g090950	Bra025311-1 Bra039065-2	HQ162473.1	AT3G27920, Myb-like protein, helps in induction of trichome development	Like Arabidopsis
GL2	KF188210	Bo6g046840	Bra003535	EU826521.1-1 EU826520.1-2	AT1G79840, Homeodomain proteinaffects trichomes initiation & development	Unknown
EGL3	KF188207-1 KF188208-2	Bo9g029230-1 Bo9g035460-2	Bra027653-1 Bra027796-2	NA	AT1G63650, a bHLH transcription factor 1, mutant has reduced trichomes	Unknown
TTG1	KF188213	Bo7g096780-1 Bo2g159360-2	Bra009770	EF175932.1-1 EF175931.1-2 EU192031.1-3 EF175929.1-4 EF175930.1-5 EU192030.1-6	AT5G24520, WD-40 protein involved in trichome development	Like Arabidopsis
TRY	KF188211-1 KF188212-2	Bo2g046050-1 Bo3g022870-2 Bo9g110930-3	Bra022637-1 Bra026297-2 Bra029089-3	EE451172(EST)	AT5G53200, Myb-like protein, mutation leads to glabrous leaves	Unknown

Genes in the same row are closest orthologues to each other. NA = Sequence not available.

### Phylogeny of B. villosa Trichome Regulatory Genes

In general, the amplified *B. villosa* translated coding sequences were over 90% identical to other *Brassica* orthologues, especially GL2 and TTG1 (Table S1), with only a few exceptions. *B. villosa* GL1 was only 82% similar to *B. rapa* GL1-2 and 75% similar to AtGL3. *B. villosa* EGL3-1 was 87% similar to *B. oleracea*, *B. rapa*, and Arabidopsis EGL3-2, while *B. villosa* EGL3-2 was 82% similar to the three latter sequences. *B. villosa* TRY-1 and TRY-2 were 86% and 87% similar to *B. oleracea* TRY-3 and *B. rapa* TRY-1, respectively. In contrast, *B. villosa* TTG1-1 and all other orthologues were only 48% similar to *B. oleracea* TTG1-2.

**Figure 1:**
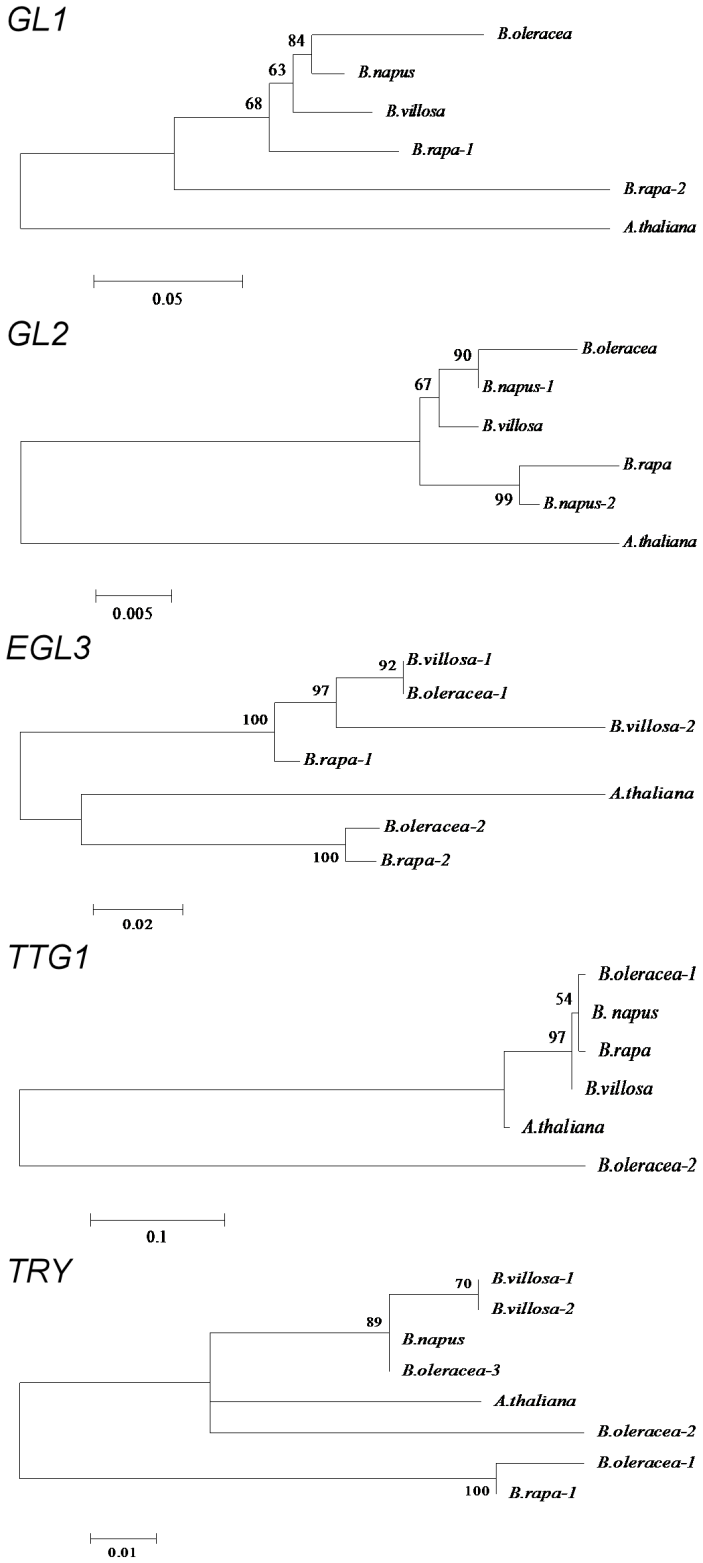
Phylogenetic relationships for the five major trichome regulatory genes present in *Brassica* and *A. thaliana*. Sequences were analysed by the maximum likelihood method with bootstrap values (%) indicated (100% is implicit in vacant branching positions). Scale indicates amino acid substitutions per site. A consensus sequence based on six *B. napus* TTG1 copies in NCBI was used for more robust analysis rather than the individual BnTTG1 copies, which each gave very weak associations due to limited overlapping sequence. *B. rapa* TRY-2 and TRY-3 were also not included since their small size gave spurious weak associations of <50%. Although three copies for *TRY* exist in *B. oleracea* and *B. villosa* (Fig. 2), *TRY-3* could not be cloned from *B. villosa* cDNA due to low expression.

Phylogenetic trees using translated protein sequences indicate that GL1 and GL2 from hairy *B. villosa* are closest to several sequences from the two non-hairy species *B. napus* and *B. oleracea* (Figure 1). The two BvEGL3 sequences both showed strong relationships to EGL3-1 from *B. oleracea* and *B. rapa,* and were less close to *B. rapa* and *B. oleracea EGL3-2* sequences, which were closer to the Arabidopsis EGL3 (*B. napus* EGL3 sequence was not available). BvTTG1 sequence was intermediate between Arabidopsis and *B. napus*, BoTTG1-1, and *B. rapa* TTG1 sequences, and farthest from BoTTG1-2 (which was only 45% similar to BoTTG1-1). BvTRY-1 and BvTRY-2 paired together and fell nearest to the pairing between the *B. napus* consensus sequence and the *B. oleracea* TRY-3 sequences. Initially, all six copies (ESTs) for BnTTG1 and the short (<100 bp) *B. rapa* sequences for BrTRY2 and BrTRY3 were used in this phylogeny testing. Later (due to differences in sequence length), only the longer overlapping BnTTG1 consensus sequence and longer BrTRY1 were compared with the other orthologues, and this improved the confidence (bootstrap values) of the phylogenetic relationships.

### Total Trichome Regulatory Gene Expression in Leaves of Hairy B. villosa, Diploid Brassica, and A. thaliana, Relative to Glabrous B. napus

Alignment of the *B. villosa* nucleotide sequences with other known *Brassica* and *A. thaliana* trichome regulatory sequences in public and private databases (data not shown) indicated conserved regions from which we designed Q-PCR primers (Table 1) to compare total expression levels of the five genes relative to orthologues of glabrous *B. napus*. In a preliminary Q-PCR expression experiment, all five trichome regulatory genes were highly expressed in trichome-bearing first true leaves of *A. thaliana* (with *GL1*, *GL2*, and *EGL3* being the highest and *TTG1* and TRY being the lowest) (Figure S2, insert). In contrast, expression of these orthologues in the first leaf of *B. villosa*, *B. oleracea*, *B. rapa* and *B. napus* (set at 1) (was dramatically lower than in Arabidopsis (Figure S2). *GL1* and *GL2* transcription was highest in *B. villosa* when only the four *Brassica* species were compared. *B. villosa* expression was proportionately more similar to *B. rapa* expression (in leaves bearing a light coverage of leaf trichomes) when total relative expression patterns of each of the four positive regulatory genes were compared across the Brassica species. Moreover, the relative expression pattern was highly distinct for the two hairy *Brassica* species compared with the glabrous *B. oleracea* and *B. napus*. Both hairy lines showed proportionately lower total leaf expression for *EGL3* and *TTG1* than *GL1* and *GL2*, but *TRY* expression in *B. rapa* was much lower. In contrast, *B. villosa* with its dense leaf trichome coverage showed high expression for *TRY*.

### Copy-specific Trichome Regulatory Gene Expression in B. villosa and its C Genome Relative, B. oleracea

**Figure 2:**
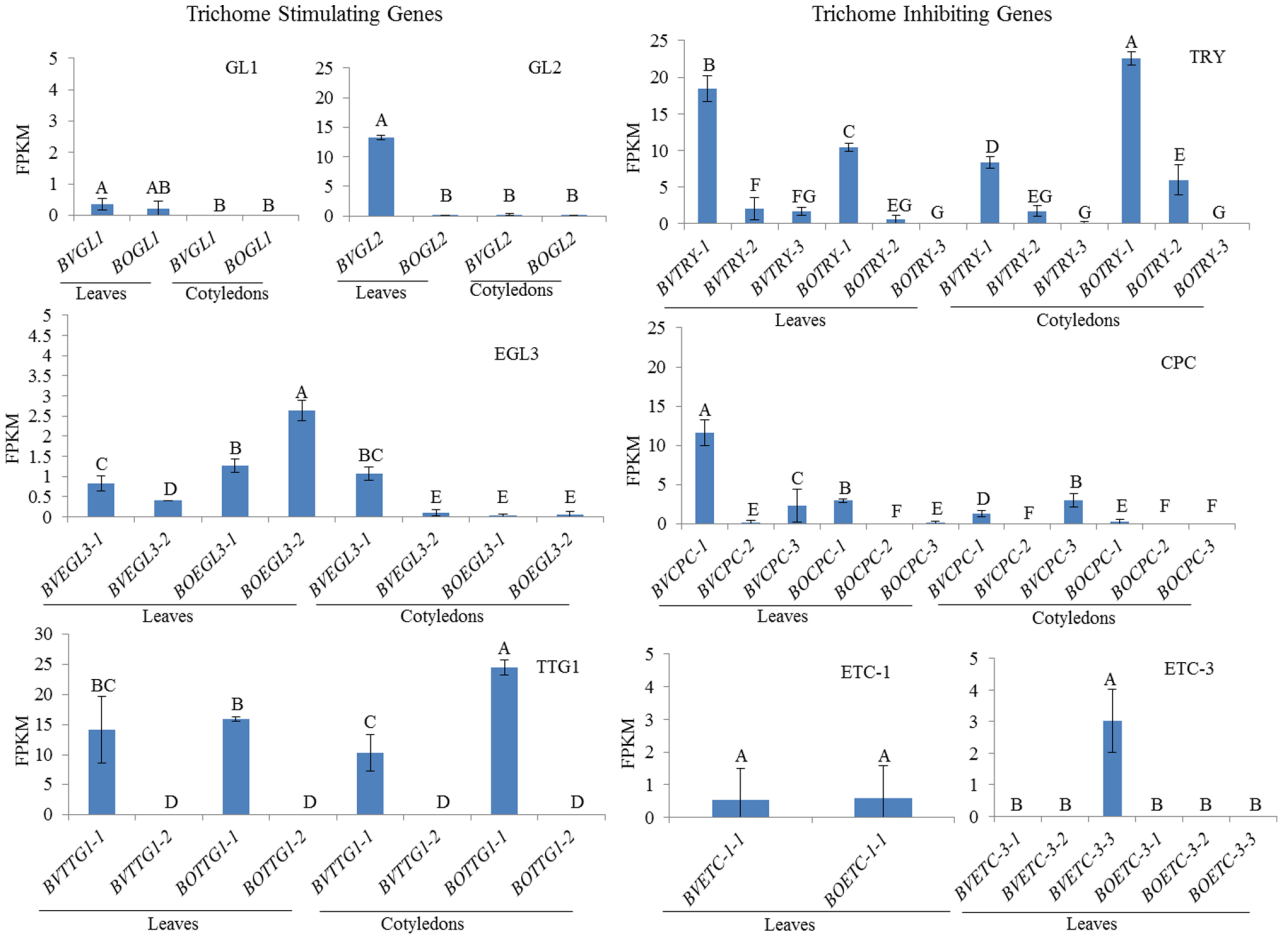
Transcript levels for individual gene copies of the four trichome positive regulatory genes and four negative regulatory genes in *B. villosa* compared with *B. oleracea*. RNAseq data is expressed as fragments per kilobase of exon per million fragments mapped (FPKM). Within each panel, different letters represent significantly different means (± standard error) for 3 independent RNA extractions (1^st^ true leaves or cotyledons from up to 10 plants per extraction) at p≤0.05.

**Figure 3:**
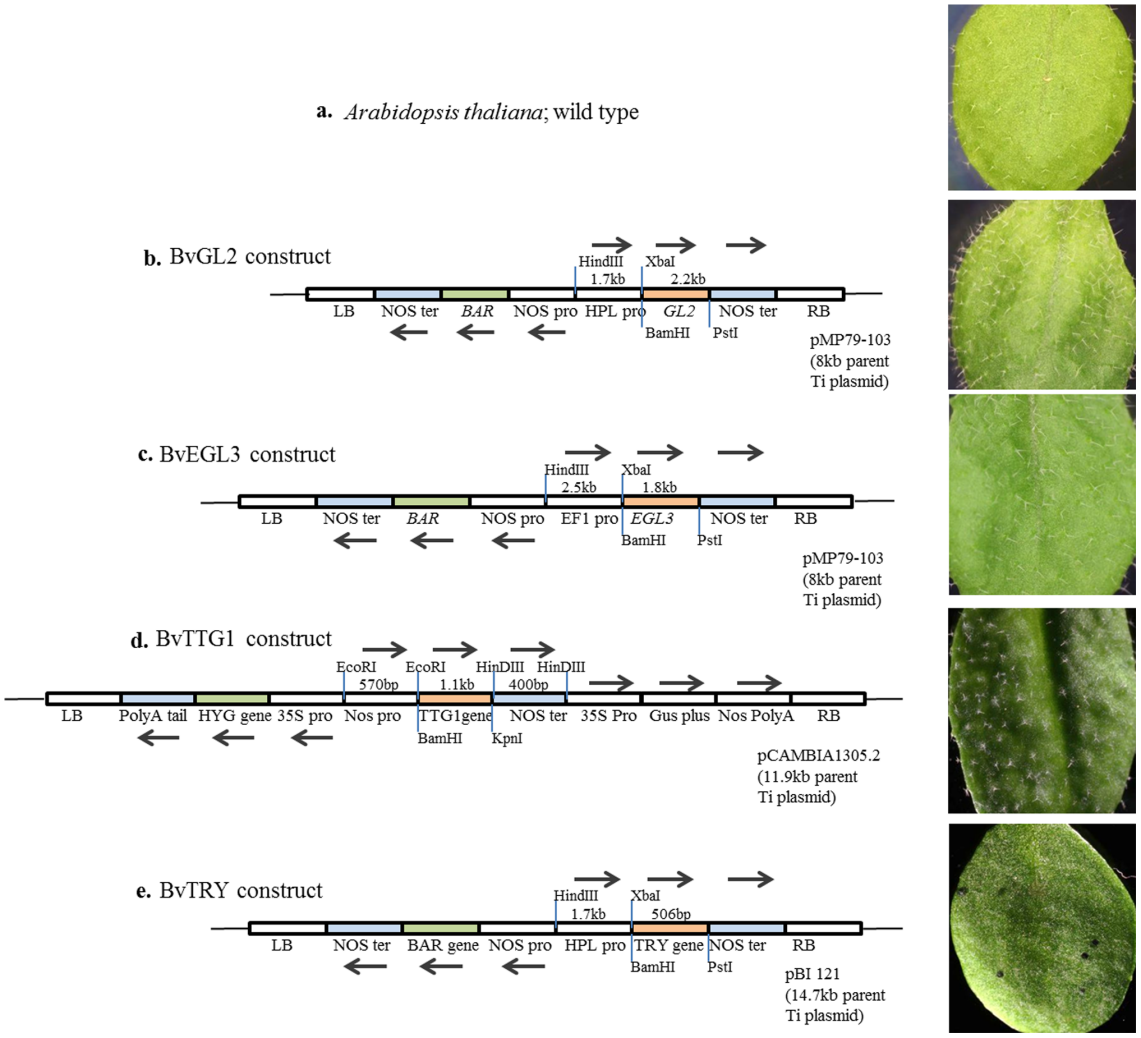
Trichome phenotypes of Arabidopsis transgenic plants overexpressing trichome related genes from *Brassica villosa*. a. Expanded leaf of wild type Arabidopsis (Columbia) showing normal trichome pattern. b. BvGL2 over-expressed transgenic leaf showing increased trichome number. c. BvEGL3 over-expressed transgenic leaf showing increased trichome number. d. BvTTG1 over-expressed transgenic leaf showing increased trichome number. e. BvTRY over-expressed transgenic leaf showing glabrous phenotype. (All photographs were taken with 10X magnification by a compound microscope at 3 weeks after germination).

Since total overall transcript levels for *GL1*, *GL2* and unexpectedly for *TRY* were very high in *B. villosa* leaves relative to the other *Brassica* species in the Q-PCR analysis, expression levels were examined for the four trichome positive regulatory genes, and *TRY* by RNA seq. This analyses was expanded to include three other major negative regulatory genes (*CPC*, *ETC1* and *ETC3*) to determine whether any particular copy was more prominently expressed in hairy *B. villosa* first true leaves (and glabrous cotyledons). To do this, we took advantage of a new reference genome in the glabrous C genome relative, *B. oleracea* (Parkin, unpublished). Expression of two copies were detected for *EGL3* and *TTG1* and three copies for *TRY* and *CPC* in *B. villosa* by mapping to the *B. oleracea* genome. RNA seq showed that the single-copy *BvGL2* gene and *BvTTG1-1* and *BoTTG1* have very high transcript levels in hairy *B. villosa* leaves compared with the *GL1* and *EGL3* positive regulatory genes. Transcripts of the single *BoGL2* were undetectable in glabrous *B. oleracea* leaves and similarly *TTG1-2* was undetectable in glabrous cotyledons of both species (Figure 2). Expression of the single copy *GL1* gene was very low (<0.5 FPKM) in leaves and equivalent between the two species, and undetectable in cotyledons. Both copies of *EGL3* were expressed at a slightly higher levels in leaves (ranging from 0.3 to 2.5 FPKM) than cotyledons (ranging from 0.05 to 1 FPKM), but the transcript levels were reduced in *B. villosa* leaves compared with *B. oleracea* leaves (Figure 2). Curiously, *BvEGL3-1* had higher leaf transcript levels proportionally to *BvEGL3-2* in *B. villosa*, whereas the converse was true in *B. oleracea*. In cotyledons, *BvEGL3-1* had expression (equivalent to leaf), and both genes were almost undetectable in *B. oleracea* cotyledons (Figure 2). The single copy *B.villosa GL3* has a similar pattern of expression to that of *BvEGL3-1*, with higher expression of *BvGL3* in cotyledons compared to *BoGL3*, whereas higher expression of *BoGL3* in true leaves compared to *BvGL3* (data not shown). The *B. villosa* single copy of *GL2*, copy 1 of *EGL3* and *TTG1* all proved to be functional proteins, since their binary expression constructs enhanced trichome density in Arabidopsis (Figure 3). Preliminary analysis of knockdown lines for these genes in both Arabidopsis and *B. napus* resulted in no observed phenotype changes (Nayidu and Gruber, unpublished).

Transcripts for three different copies of the *TRY* negative regulatory gene and two copies of *BoTRY* were detected (Figure 2). Very high leaf and cotyledon expression was detected for *BvTRY-1* in *B. villosa* (18–20 FPKM) and *BoTRY-1* in *B. oleracea* (9–10 FPKM), while *BvTRY-2/BoTRY-2* and *BvTRY-3/BoTRY-3* transcript levels were much lower in both leaves and cotyledons (Figure 2). The ranking and proportionate expression of individual *TRY* gene copies for *B. oleracea* was similar to *B. villosa*, but 50% lower in leaves and much higher in cotyledons. The higher combined expression level (FPKM value) for the three *TRY* genes in trichome-rich *B. villosa* leaves correlated with the overall higher total *TRY* expression level measured in the *B. villosa* leaves by Q-PCR. The fact that the relative expression for all three *TRY* genes was quite similar in both species and that *BvTRY-1* and *BoTRY-1* expression was equally high regardless of the extreme differences in leaf trichome density between *B. villosa* and *B. oleracea* was inconsistent with the Arabidopsis model of *TRY* being a negative regulator in *B. villosa*, even though Arabidopsis leaf trichomes were eliminated when Arabidopsis was transformed with a *BvTRY-1* expression construct (Figure 3).

Since the expression pattern of *TRY-1* was higher in trichome-covered *B. villosa* leaves, we took advantage of the new *B. oleracea* genome database to examine individual copies of several other prominent negative regulatory genes, *CPC*, *ETC1*, and *ETC3* ([23]; Table 2). Only one copy existed for *ETC1* in both *B. villosa* and *B. oleracea* and the expression level of both was very low (0.05 FPKM) in leaves (Figure 2) and undetected in cotyledons of both species (data not shown). *ETC3* had three copies in both species, but only *BvETC3-3* was expressed (2–4 FPKM) in *B. villosa* leaves (Figure 2), and the other copies were undetectable in either species and in cotyledons (data not shown). In contrast, all three copies of the *CPC* gene were expressed in leaves of both species, but expression of *CPC-1* was predominant in hairy *B. villosa* leaves and 6-fold higher than *BoTRY-1* in glabrous *B. oleracea* leaves. The other two *CPC* copies had low transcript levels in leaves and cotyledons, and *BvCPC-3* expression (2.5 FPKM) was predominant in cotyledons.

### Sequence Comparisons between B. villosa, three other Brassicas and Arabidopsis

Since gene function impacts phenotype from a combination of transcript level and translated protein sequence, amino acid sequence diversity was determined for translated sequences of the five cloned regulatory genes in *B. villosa*, the three other *Brassica* species and *A. thaliana* (Figure S1). Overall, sequences for *B. villosa* were quite similar to those of the other *Brassicas* and *A. thaliana* in length, M*r* and p*I*, with the following exceptions (Table 3). BvEGL3-2 was 20 kDa shorter than BvEGL3-1 from *B. villosa* and the other EGL3 sequences from *B. oleracea*, *B. rapa*, and Arabidopsis. The M*r* of BvTRY-1 was most similar to that of *B. oleracea*, *B. napus*, and Arabidopsis, while BvTRY-2 was smaller. Isoelectric points for proteins from *B. villosa* were similar to those of orthologues in other *Brassica* species and *Arabidopsis* and usually acidic, except in the case of GL1 (Table 3). Here, the *B. villosa* orthologue BvGL1 was neutral and had a *p*I closest to *B. rapa* BrGL1-1 rather than to alkaline pI of BnGL1-1, BoGL1-1, BrGL1-2, BoTTG1-2 and most of the TRY sequences.

**Table 3:** Theoretical protein size (Mr/Number of amino acid) and isoelectric point (*pI*) of trichome regulatory coding sequences in the *Brassicaceae*.

	*B. napus* (Mr)	*B. oleracea* (Mr)	*B. rapa* (Mr)	*B. villosa (*Mr)	*A. thaliana* (Mr)	*B. napus* (*pI*)	*B. oleracea* (*pI*)	*B. rapa* (*pI*)	*B. villosa* (*pI*)	*A. thaliana* (*pI*)
GL1-1 GL1-2	26.0/225	24.7/212	26.1/22522.7/199	26.1/225	26.3/228	8.80	9.42	7.67 9.22	7.69	6.66
GL2-1 GL2-2	83.7/75083.7/750	83.6/748	83.8/750	83.5/748	86.5/776	6.58 6.83	6.58	6.33	6.56	6.38
EGL3-1 EGL3-2	ND	64.0/57367.6/604	66.3/59667.8/606	66.5/59746.6/421	66.6/616	ND	5.59 5.15	5.47 5.14	5.52 6.20	5.06
TTG1-1 TTG1-2 TTG1-3 TTG1-4 TTG1-5 TTG1-6	37.3/337 37.2/33737.2/337 37.3/33737.4/338 37.2/337	37.2/33724.5/212	37.3/337	37.3/337	37.9/341	4.66 4.66 4.66 4.66 4.66 4.70	4.66 10.77	4.66	4.66	4.71
TRY-1 TRY-2 TRY-3	13.1/107	13.0/10713.0/10619.0/160	9.07/756.51/546.34/51	13.0/1079.02/75	13.0/106	9.58	9.519.249.02	9.299.515.28	9.219.39	9.51

ND, not determined (sequence not available). Closest orthologues between the species are positioned within the same row. Data represents all known orthologues and homologues for each species.

Many amino acid substitutions, additions and deletions were apparent within each of the five trichome genes when comparisons were made between *B. villosa* and the four other species. In total, there were 58 amino acid changes (26.2%) out of a total of 237 consensus amino acids (CAA) for GL1, 30 (3.8%) out of 786 CAA for GL2, 92 (15.1%) out of 608 CAA for EGL3-1, 132 (27.2%) out of 485 CAA for EGL3-2, and 111(31.2%) out of 356 CAA for TTG1. TRY genes were the smallest compared to the positive regulatory genes and had the most amino acid differences between the species: 99 (84.6%) out of 117 CAA for TRY-1 and 111 (69.4%) out of 160 CAA for TRY-2 (Figure S1). Out of the total amino acid differences observed, those which were unique to *B. villosa* or distinguished hairy germplasm from glabrous germplasm were more closely examined (Table 4, Figure S1). Several particularly noteworthy variations between hairy and glabrous germplasm involved more extreme charge and hydrophobicity differences that potentially could affect the molecular structure and functional properties of a protein. These included consensus amino acid (CAA) position 223 in the 3′ end of GL1, where the aromatic phenylalanine [38] in *B. villosa* and *B. rapa* and the hydrophobic residue leucine (Leu) in *A. thaliana* and *B. rapa* [hydrophobicity values of 2.8 (F) and 3.8 (L), respectively [39]] were replaced by the slightly hydrophilic serine (Ser, value of −0.8) in glabrous *B. napus* and *B. oleracea* (Table 4). CAA position 273 in GL2 included a glutamine (Gln) residue in hairy *B. villosa*, *B. napus* (one out of two copies), and the hairy *B. rapa* sequences, and a negatively charged glutamate acid (Glu) residue in *A. thaliana*. These were replaced by a positively charged imidazole ring (histidine, His) in the other copy of glabrous *B. napus* and in glabrous *B. oleracea* sequences. CAA position 439 in GL2-1 included an alanine conserved between hairy *B. villosa*, *A. thaliana*, and *B*. *rapa* proteins and replaced by a hydrophobic valine in GL2 of glabrous *B. napus* and *B. oleracea*. Finally, three positions in BvGL1, two in BvGL2, two in EGL3, one in BvTTG1, and one in TRY were unique to *B. villosa*. In particularly, the serine in CAA position 154 of all BvTRY sequences was replaced by an arginine in TRY genes from all other species.

**Table 4:** Specific amino acids in five trichome regulatory genes within the *Brassicaceae*.

	*Consensus position	*A. thaliana* hairy	*B. rapa* AA hairy	*B. napus* AC glabrous	*B.oleracea* CC glabrous	*B. villosa* CC hairy
GL1	137*	Pro	Pro	Pro	Pro	Thr
	154*	Gln	Gln	Gln	Gln	Glu
	202*	Asn	Asn	Asn	Asn	Asp
	223(1) 223(2)	Leu NA	Phe Leu	Ser NA	Ser NA	Phe NA
GL2	273(1) 273(2)	Glu NA	Gln NA	His Gln	His NA	Gln NA
	282*	Tyr	Tyr	Tyr	Tyr	Phe
	287*	Ala	Ala	Ala	Ala	Ser
	439-(1) 439-(2)	Ala	Ala	Val	Val	Ala
EGL3	171(1) * 171(2)	Val NA	Val Val	ND ND	Val Val	Ala Val
	552*	Leu	Leu	ND	Leu	Val
TTG1	4*	Ser	Ser	Ser	Ser	Ala
TRY	154(1) * 154(2) * 154(3) *	Arg NA NA	Arg NIL NIL	Arg NA NA	Arg Arg Arg	Ser Ser NA

*Selected positions in the aligned consensus amino acid sequence (CAA) were selected from Figure S1 if they distinguished hairy from glabrous germplasm (dark arrows in Fig. S1) or were unique to *B. villosa* (*red arrows in Fig. S1). ND, not determined (sequence not available). NIL, missing amino acid. NA, not applicable. Note: Multiple gene copies are only indicated if an amino acid differed between the copies.

Each of the four trichome initiation genes and TRY had domains which were conserved within all the *Brassica* species and *A. thaliana*, but they also showed species distinctions not correlated with a trichome phenotype (Figure S1). GL1 consisted of well known conserved R2 and R3 MYB DNA binding domains, but *B. rapa* GL1 was missing CAAs 1-29 and the *B*o*GL1* had a nonsense mutation at the end of the sequence and five unique amino acids (CAA position 119–123) in a conserved region immediately downstream of the R3MYB domain. GL2 showed no significant differences in the conserved homeobox domain and had the most consistent amino acid profile across all species compared to the other regulatory genes (Figure S3). However, CAA positions 114 to 118 present in the conserved *A. thaliana* START domain and the unique 29 amino acid (aa) Arabidopsis leader sequence were missing in GL2 from the four *Brassica* species (Figure S1). EGL3 translated sequences were mainly equivalent in their bHLH-DNA binding domains, but variable between CAA 391-3 in all orthologues (Figure S1). Noteworthy was an entirely different sequence post-CAA 426 for *B. villosa* EGL3-2, whereas all other EGL3s were missing aa from 426–455 and 485 to the stop codon, aa 456–484 were completely different, and sequences for *B. oleracea* EGL3s were missing CAAs 164–187. BoTTG1-2 and TRY also showed diversity. TRY was completely conserved in the R3 MYB binding domain but diverse in other areas (Figure S1). BvTRY-2, BrTRY-1 and BrTRY-2 sequences were each missing CAA 53–85, *B. rapa* TRY-3 was missing aa 105–160 within and beyond the R3 binding region, TRY-3 of *B. oleracea* had a unique 52 aa leader sequence, while *B. rapa* TRY-2 and 3 had the smallest R3 MYB binding regions. Except for CAA 1–85 missing in BvTRY-2, BvTRY-1 and BvTRY-2 were identical, but the very low expression of *B*v*TRY-3* prevented evaluation.

### Ka/Ks Amino Acid Substitutions

Leucine (L), serine (S) and arginine (R) were the most abundant amino acids within all five trichome regulatory genes for *B. villosa* as well as the other four species (Figure S3). However, cysteine was completely absent in TRY orthologues (except for *B. oleracea* and *B. rapa* TRY-2, each of which had one cysteine at CAA 95). Moreover, *B. villosa* TRY-2 and *B. rapa* TRY-1 were under-represented in V, F, S, T, D, H, K, and R compared with BvTRY-1 and TRY in other species examined (Figure S3). This led us to determine *Ka*/*Ks* amino acid substitution values for the five trichome genes to assess whether sufficiently different amino acid substitutions had occurred to potentially change protein function.

*Ka*/*Ks* values were much less than one for most pair-wise comparisons between homologues and orthologues, especially for GL2, TTG1, and TRY, indicating mainly synonymous substitutions that would not impact protein structure (Table S1). *Ka*/*Ks* values for EGL3 sequence comparisons were higher in general than values for GL2, TTG1, and TRY comparisons, but less than one and lower than many of the *Ka*/*Ks* values determined for GL1 sequences (Table 5; Table S1). Most pairwise comparisons between GL1 in *B. villosa* and each *Brassica* species and Arabidopsis consistently showed synonymous substitutions. However, *Ka*/*Ks* ratios for BvGL1 and BoGL1 were much closer to one, indicating an evolutionary tendency towards significant change. This was even more extreme between GL1 from *B. villosa* and *B. rapa* (1.04) and from *B. villosa* and *B. napus* (1.87) (Table 5), indicating large substitution differences that may have the potential for functional changes.

**Table 5:** *Ka*/*Ks* * ratios for trichome regulatory gene comparisons between *B. villosa* and three other *Brassica* species and Arabidopsis.

Pairwise Comparison		GL1	GL2	EGL3	TTG1	TRY
*B. napus*	*B. villosa*	1.87	0.09	NA	0.03	0.23
*B. villosa*	*B. rapa-1*	1.04	0.06	0.42	0.02	0.19
*B. oleracea*	*B. villosa*	0.78	0.14	0.51	0.1	0.06
*B. villosa*	*B. rapa-2*	0.21	–	0.31	–	–+
*B.villosa*	*A. thaliana*	0.24	0.07	0.2	0.01	0.06
*B.napus-2*	*B.villosa*	0.09	0.09	0.09	0.09	–
*B.villosa-1*	*B.villosa-2*	–	–	0.86	–	0.00
*B.villosa-1*	*B.oleracea-2*	–	–	0.30	0.27	0.15
*B.oleracea-1*	*B.villosa-2*	–	–	0.86	–	0.06
*B.villosa-2*	*B.oleracea-2*	–	–	0.40	–	0.15
*B.villosa-2*	*B.rapa-2*	–	–	0.49	–	–+
*B.villosa-2*	*A.thaliana*	–	–	0.21	–	0.06
*B.napus-3*	*B.villosa*	–	–	–	0.05	–
*B.napus-4*	*B.villosa*	–	–	–	0.03	–
*B.napus-5*	*B.villosa*	–	–	–	0.03	–
*B.napus-6*	*B.villosa*	–	–	–	0.03	–
*B.villosa-1*	*B.oleracea-3*	–	–	–	–	0.19
*B.villosa-2*	*B.oleracea-3*	–	–	–	–	0.19

*Ka/*K_S_* (Yang Z, 1997): *Ka*, non-synonymous nucleotide substitution. *Ks*, synonymous nucleotide substitution value.

+ *B. rapa*-2 (BRTRY-2) and *B. rapa*-3 (BRTRY-3) amino acid sequences were too short to be included. NA, *B. napus* sequence not available.

## Discussion

*Brassica villosa* is a weedy C genome species of the *Brassicaceae* and is of interest to molecular evolutionists and plant breeders for its dense trichome patterning on most tissues [33]. In the present study, we compared expression patterns and coding sequences for key trichome regulatory genes of *B. villosa* with those of orthologues from *A. thaliana* and several species within *Brassica* A and C genomes, as a means of distinguishing unique *B. villosa* sequence patterns from those of more modest trichome-bearing species and glabrous germplasm. Phylogenetic trees indicated that GL1, GL2, EGL3 and TRY sequences from hairy *B. villosa* are closest to several orthologues from the two non-hairy species *B. napus* and *B. oleracea.* These data confirm the closer relationship of these *B. villosa* positive regulatory trichome genes to those of *B. oleracea* and the C genome of *B. napus* than to Arabidopsis regardless of the trichome density differences. Li and co-workers showed pairing of GL1 sequences from hairy *Brassica incana* (related to *B. villosa*) with those from non-hairy *B. napus* lines (with a 92–94% sequence identity) [29].

Generally, RNA sequencing showed that transcript levels of one of the multiple copies of *EGL3*, *TTG1*, *TRY*, *CPC*, and *ETC3* were predominant compared to levels of the other copies in *B. villosa*. Expression for the trichome stimulating genes, *BvGL2* and *BvTTG1*, and the trichome inhibiting genes, *BvTRY-1* and *BvCPC-1*, was very high in young true leaves of *B. villosa* (where dense trichome coverage is found) and low in cotyledons compared with *GL1*, *EGL3*, *ETC1*, and *ETC3* genes. This *B. villosa* RNAseq pattern was distinct and somewhat unexpected since the *BvEGL3* expression was lower than in glabrous *B. oleracea* leaves and the *BvTTG1* expression was quite similar in level to *BoTTG1* expression. Expression of the *BvTRY-1* and *BvCPC-1* was also high in hairy *B. villosa*, and expression of *BoTRY-1* was high in glabrous *B. oleracea* leaves. This was inconsistent with the Arabidopsis model of TRY and CPC as negative regulators of trichome initiation, where enhanced leaf trichome density phenotype occurred when TRY expression was knocked down in Arabidopsis *try* mutants [40]. The data implies that *BvTRY-1, BvCPC-1,* and (potentially) *BvETC3-1* genes may not behave as negative regulators of trichome initiation in *B. villosa.* Protein coding sequences for BvTRY-1 and BvTRY-2 genes were closest to those of non-hairy BoTRY-3 and BnTRY, and all four of these are closer to each other and to *A. thaliana* than to the other *Brassica* TRY genes. Redundant trichome negative regulatory genes exist in the *A. thaliana* model [25] and functional redundancy can speed up gene evolution [41]–[43]. Hence, *B villosa* may use these R3 regulatory genes with high expression for a different purpose in the densely covered *B. villosa* true leaves. This hypothesis is supported by the insertion of *BvTRY-1* into *B. napus*, yielding transgenic plants in which trichome density is not affected even though the same binary construct was used to depress Arabidopsis trichome development (Nayidu and Gruber, unpublished and Figure 3 respectively). In the future, it will be particularly useful to express these *B villosa* genes in a range of other Brassica species and to develop knock-out RNAi lines in *B. villosa* to solve the mystery of their true function. This will depend on the development of a transformation system for *B. villosa*, such as the protocols that now exist for *B. napus*, *B. rapa*, *B. oleracea,* and *B. carinata* [44]–[47]. Additional analysis of *B. villosa* gene structure is also necessary for a complete understanding of their introns, untranslated regions, and promoters. For example, intron 1 and 30 non-coding nucleotides in *A. thaliana* are important for the expression of the *GL1* gene [5], [48], [49]. A 620 bp fragment of the *TRY* promoter contains sequences that mediate the repression of its own expression, and deletion of this promoter region can rescue the *A. thaliana try* mutant phenotype [50]. Moreover, the Arabidopsis TRY promoter contains sequences regulated by microRNA-regulated “*SQUAMOSA PROMOTER BINDING PROTEIN LIKE*” (*SPL*) genes [50], and enhanced expression of the SPL-regulator miR156b in Arabidopsis increases trichome density [51].

The high expression pattern for GL2 and TRY-1 in *B. villosa* leaves led us to compare the coding sequences of the five trichome regulatory genes we had isolated from *B. villosa* with those in *B. napus*, two diploid *Brassica* species, and *A. thaliana*. Extreme trichome coverage in *B. villosa* leaves may be due (at least in part) to polymorphism and evolutionary differences that impact on protein function rather than solely to specific gene transcript levels, which are dictated by promoter “strength” and intra-gene regulatory structures. Although analysis showed few differences between most orthologues, overall evolutionary selection was detected between GL1 proteins of hairy (*B. villosa*, *B. rapa*) and glabrous (*B. napus*) genotypes by their high pairwise *Ka*/*Ks* values (>1), potentially due to sites involved in adaptive change. Adaptive changes may occur at surprisingly few sites; consequently, the overall *Ka*/*Ks* ratio for an entire protein may remain dominated by non-adaptive changes and be substantially lower than unity (reviewed in [52]) as seen by the *Ka*/*Ks* scores for *GL2*, *EGL3*, *TTG1*, and TRY. Moreover, once a protein achieves a new advantageous function, the frequency of non-synonymous substitutions at the adapted sites will be reduced by new functional constraints [52].

A substantial proportion of the differences that distinguished the four *Brassica* trichome regulatory sequences occurred outside of conserved domains. Several of these individual amino acid differences were sufficiently dramatic to potentially change the molecular and functional properties of these proteins. Particularly, three variable positions in GL1 and GL2 translated sequences distinguished hairy *B. villosa* and *B. rapa* from glabrous *B. napus* and *B. oleracea* germplasm, and all the *B. villosa* genes had a minimum of one unique site (and usually more) compared with the other species. Bloomer [53] reported on the effects of natural variation in GL1 from Arabidopsis and suggested that qualitative differences in trichome phenotypes (glabrous or hairy) might have arisen independently several times by three unique protein coding changes and a whole locus deletion. The same authors also suggested that quantitative variation (in trichome density) might have arisen because of completely linked amino acid replacements and mutations in a known (as yet uncharacterized) enhancer region within the *AtGL1* locus. In addition, Li et al. proposed that a 5-bp deletion in exon 3 of the *B. rapa GL1* gene (starting at CAA 110 in the present study) is the basis of a hairless phenotype that arose from a normally hairy double haploid brown-seeded line [29]. Hence, the extremely dense trichome coverage of *B. villosa* could be due to a combination of relatively higher transcription of GL2, hydrophobic amino acids and evolutionary changes in GL1, as well as the replacement of serine in all BvTRY sequences, and potentially a different function for TRY-1, CPC-1, and ETC3-3 (consistent with their higher *B. villosa* leaf transcript levels). Moreover, the glabrous leaves of the C genome relative *B. oleracea* could result from two non-synonymous substitutions (from asparagine to serine (at CAA 26 and 112), five continuous amino acid replacements (at CAA 119–123), one nonsense mutation (at CAA 224) leading to a shortened GL1 amino acid sequence, and a missing aa stretch in the bHLH DNA binding region of BoEGL3-1 and BoEGL3-2. Our study adds additional sequence variation data to a previous report detailing two 1 bp deletions and 1 bp insertion in exon 3 of the *B. oleracea* GL1 sequence [29]. Changes in protein function with individual amino acid modifications are also seen in other species and genes. Yeam et al. (2007) showed that a G/R polymorphism at aa 107 of the *Capsicum* eukaryotic translation initiation factor 4E protein (eIF4E) is sufficient for the acquisition of resistance against several *Capsicum* and tobacco etch potyvirus strains by expressing the amino acid substituted gene in potato (*Solanum lycopersicum*) [54].

Likely, the genetic variation we have uncovered is the “tip of the iceberg” in terms of variation that affects the function of *Brassica* trichome genes, since the trichome pathway in the simpler *A. thaliana* genome is already considered to be an integrated hierarchy of regulation by complex cell cycle status, transcriptional control and cytoskeletal function [55]. This is confirmed by the ever increasing number of trichome genes being discovered in *A. thaliana* mutant populations (>80 genes known in TAIR) [38] (Taheri et al., 2014 submitted manuscript from the same lab) and by the on-going discovery of *cis*-regulatory sequences that provide greater diversification in gene function, eg. for GL1 and MYB23 [56]. The constant improvement in genomic-scale sequencing technology, SNP analysis, and computation can now be applied to characterize large within-accession and within-species variance in trichome gene patterning in the *Brassicaceae* and identify statistically robust associations hitherto undetectable [57], [58].

## Conclusion

The present study utilized expression profiling and bioinformatics to compare *B. villosa* trichome regulatory genes with their orthologues in *B. oleracea, B. rapa, B napus,* and *A. thaliana*. In doing so, we discovered the potential for positive evolutionary selection in the *GL1* gene between *B*. *villosa*, *B*. *rapa* and *B*. *napus*. Several point mutations found within GL1 and GL2 protein sequences correlated with hairy and glabrous phenotypes within these five species and have the potential to be predictive factors. We also discovered high transcript levels for *BvGL2*, *BvTTG1*, *BvTRY-1*, and *BvCPC-1* in *B. villosa* leaves with densely covered trichomes. This *B. villosa* gene expression pattern is contrary to the trichome gene models in Arabidopsis in which cells are glabrous when expression of trichome R3 MYB inhibitor genes is high and trichomes are present in greater density and clustered when these genes are knocked down by mutation [23]. These *B. villosa* genes are now being tested in comparative over-expression studies in Arabidopsis and *B. napus*. Investigations on these genes should also be expanded to include a much broader range of hairy and glabrous *Brassica* germplasm within the Triangle of U [26].

## Supporting Information

Figure S1 Alignment of translated protein sequences for four major trichome positive regulatory genes and one negative regulatory gene in the *Brassicaceae*. GL1: Conserved R2 and R3 MYB DNA binding domains are boxed. A unique 5 aa variable sequence and a unique 14 aa 3′ deleted sequence are designated in the *B. oleracea* sequence by ovals. GL2: The conserved HDZip homeobox domain and a large START domain are boxed. A unique *A. thaliana* 29 aa leader sequence is delineated by an oval. GL3: The conserved bHLH protein binding domain is boxed. TTG1: Two conserved WD40 protein domains are boxed. A unique 44 aa leader sequence in *B. oleracea* is designated by an oval. TRY: The conserved R2 MYB DNA binding domains are boxed. A 33 aa unique deletion in the *B. rapa* gene is designated by an oval. For all 5 genes, unique amino acid modifications are designated for *B. villosa* (red arrows) and for hairy vs. glabrous plants (blue arrows).
(PDF)

Figure S2 Relative expression of four trichome regulatory genes and one negative regulatory gene in leaves of hairy *Brassica* villosa, three other Brassica species, and *A. thaliana*. Main panel shows expressed transcripts from four *Brassica* species. Insert panel shows *A. thaliana* orthologues which are much more highly expressed compared with the *Brassica* species. Expression (Q-PCR) in both panels is relative to glabrous *B. napus* cv. Westar (set at 1). Different letters in both panels indicate significant differences of the means (± standard error) at p≤0.05 in an LSD test (SAS, 2008).
(TIF)

Figure S3 Amino acid profiles for five major trichome regulatory sequences from Brassica villosa, three other *Brassica* species, and *A. thaliana*.
(TIF)

Table S1 *Ka/Ks values from pairwise comparisons of all trichome regulatory gene orthologues and homologues within four Brassica species and Arabidopsis.
(DOCX)
