# Supplementary material for: Comparison of Five Major Trichome Regulatory Genes in Brassica villosa with Orthologues within the Brassicaceae
Source: PLoS One. 2014 Apr 22;9(4):e95877. doi: 10.1371/journal.pone.0095877 (PMC3995807; doi:10.1371/journal.pone.0095877)
Supplement: Figure S1 — Alignment of translated protein sequences for four major trichome positive regulatory genes and one negative regulatory gene in the Brassicaceae. GL1: Conserved R2 and R3 MYB DNA binding domains are boxed. A unique 5 aa variable sequence and a unique 14 aa 3′ deleted sequence are designated in the B. oleracea sequence by ovals. GL2: The conserved HDZip homeobox domain and a large START domain are boxed. A unique A. thaliana 29 aa leader sequence is delineated by an oval. GL3: The conserved bHLH protein binding domain is boxed. TTG1: Two conserved WD40 protein domains are boxed. A unique 44 aa leader sequence in B. oleracea is designated by an oval. TRY: The conserved R2 MYB DNA binding domains are boxed. A 33 aa unique deletion in the B. rapa gene is designated by an oval. For all 5 genes, unique amino acid modifications are designated for B. villosa (red arrows) and for hairy vs. glabrous plants (blue arrows). (PDF) [file pone.0095877.s001.pdf]

Figure S1. Alignment of translated protein sequences from known orthologoues of five trichome regulatory genes (GL1, GL2, EGL3, TTG1, TRY) in the *Brassicas* and Arabidopsis

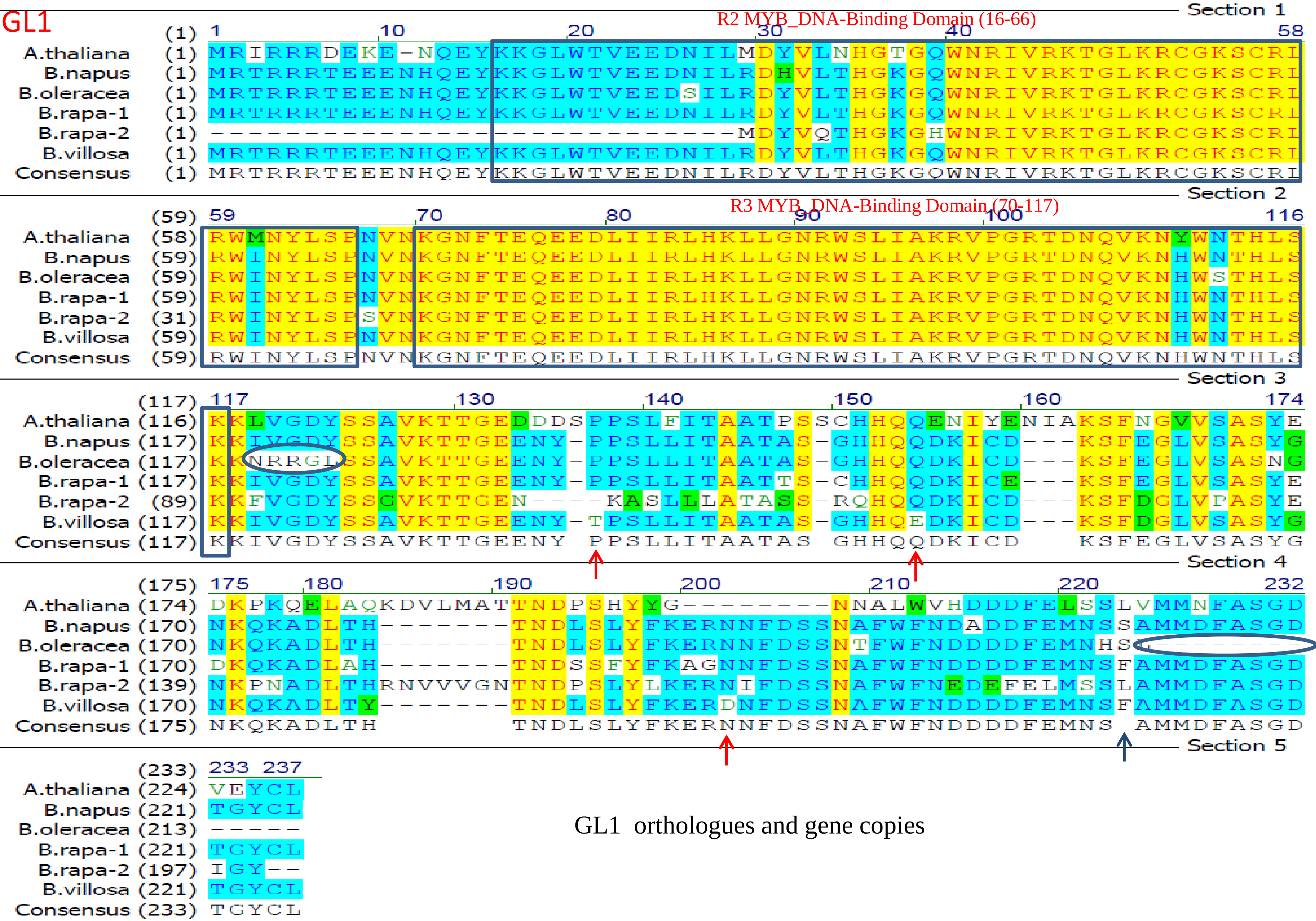

GL1 orthologues and gene copies

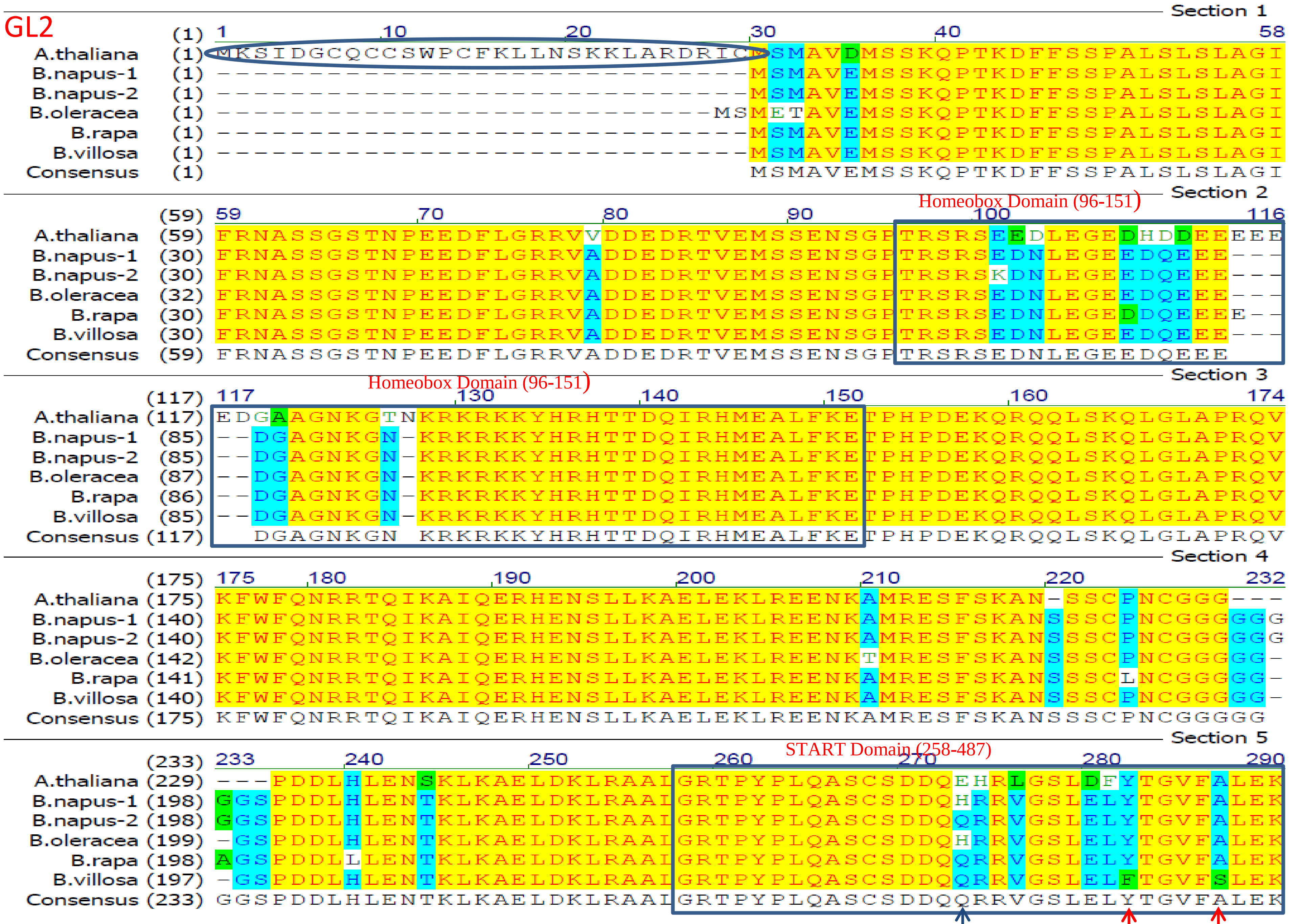

GL2 orthologues and gene copies

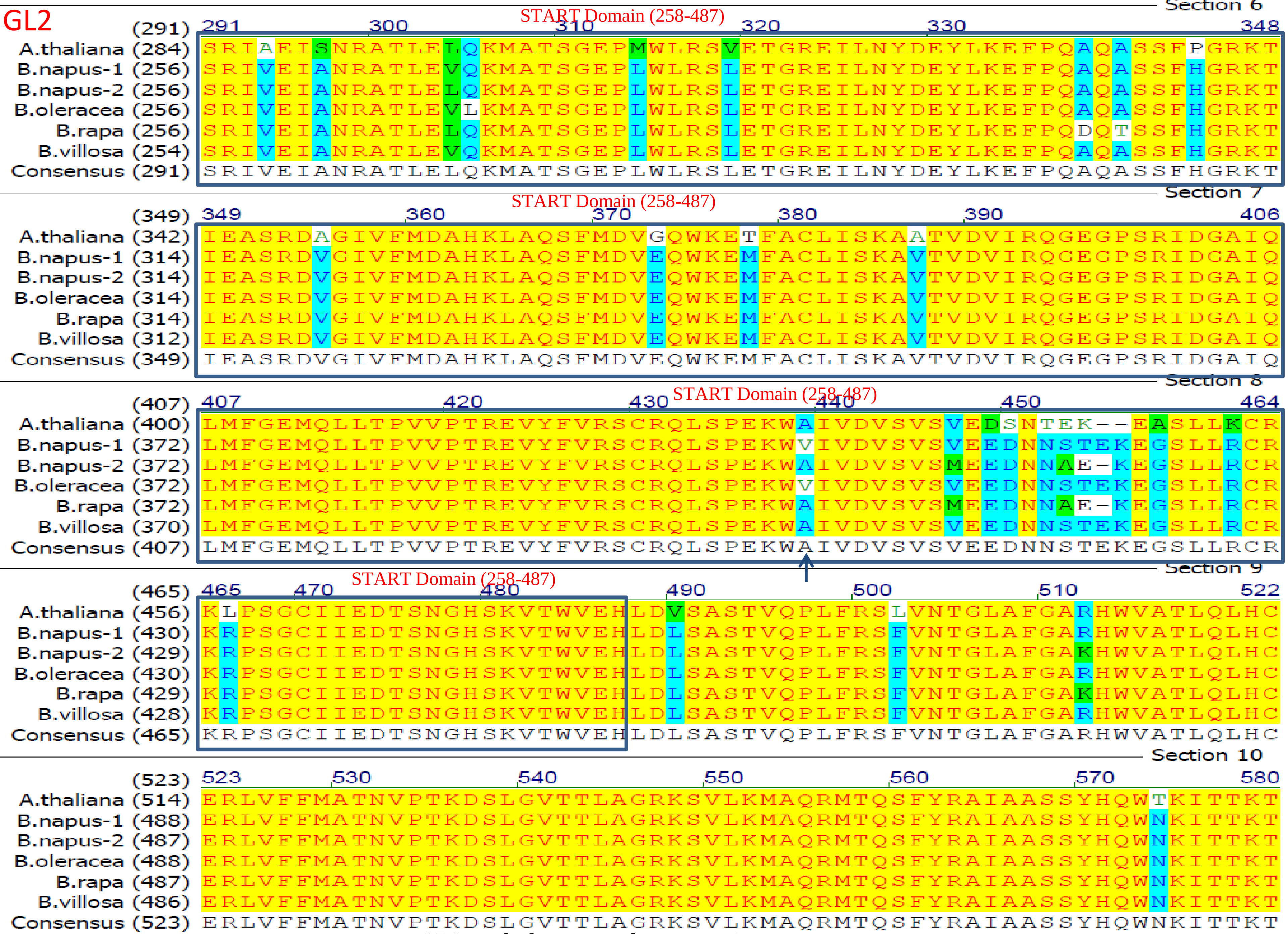

GL2 orthologues and gene copies

GL2

Section 11

```
            (581) 581                590                 600                  610                 620               638
A.thaliana  (572) GQDMRVSSRKNLHDPGEPTGVIVCASSSLWLPVSPALLFDFFRDEARRHEWDALSNGA
B.napus-1   (546) GQDMRVSSRKNLHDPGEPTGVIVCASSSLWLPVSPTLLFDFFRDETRRHEWDALSNGA
B.napus-2   (545) GQDMRVSSRKNLHDPGEPTGVIVCASSSLWLPVSPTLLFDFFRDETRRHEWDALSNGA
B.oleracea  (546) GQDMRVSSRKNLHDPGEPTGVIVCASSSLWLPVSPTLLFDFFRDETRRHEWDALSNGA
B.rapa      (545) GQDMRVSSRKNLHDPGEPTGVIVCASSSLWLPVSPTLLFDFFRDETRRHEWDALSNGA
B.villosa   (544) GQDMRVSSRKNLHDPGEPTGVIVCASSSLWLPVSPTLLFDFFRDETRRHEWDALSNGA
Consensus   (581) GQDMRVSSRKNLHDPGEPTGVIVCASSSLWLPVSPTLLFDFFRDETRRHEWDALSNGA
```

Section 12

```
            (639) 639                650                 660                  670                 680               696
A.thaliana  (630) HVQSIANLSKGQDRGNSVAIQTVKSREKSIWVLQDSSTNSYESVVVYAPVDINTTQLV
B.napus-1   (604) HVQSIVSLSKGQDRGNSVSIQTVKSREKSTWVLQDSCTNSYESVVVYAPVDINTTQLV
B.napus-2   (603) HVQSIVSLSKGQDRGNSVSIQTVKSREKSTWVLQDSCTNSYESVVVYAPVDINTTQMV
B.oleracea  (604) HVQSIVSLSKGQDRGNSVSIQTVKSREKSTWVLQDSCTNSYESVVVYAPVDINTTQLV
B.rapa      (603) HVQSIVSLSKGQDRGNSVSIQTVKSREKSTWVLQDSCTNSYESVVVYAPVDINTTQMV
B.villosa   (602) HVQSIVSLSKGQDRGNSVSIQTVKSREKSTWVLQDSCTNSYESVVVYAPVDINTTQLV
Consensus   (639) HVQSIVSLSKGQDRGNSVSIQTVKSREKSTWVLQDSCTNSYESVVVYAPVDINTTQLV
```

Section 13

```
            (697) 697                710                 720                  730                 740               754
A.thaliana  (688) LAGHDPSNIQILPSGFSIIPDGVESRPLVITSTQD-DRNSQGGSLLTLALQTLINPSP
B.napus-1   (662) IAGHDPSNIQILPCGFSIIPDGVESRQLVISSAQE-DRNTQGGSLLTLALQTLVNQSP
B.napus-2   (661) IAGHDPSNIQILPCGFSIIPDGVESRQLVISSAQEADRNTQGGSLLTMALQTLVNQSP
B.oleracea  (662) IAGHDPSNIQILPCGFSIIPDGVESRQLVISSAQE-DKNTQGGSLLTLALQTLVNQSP
B.rapa      (661) IAGHDPSNIQILPCGFSIIPDGVESRQLVISSAQEADRNTQGGSLLTMALQTLVNQSP
B.villosa   (660) IAGHDPSNIQILPCGFSIIPDGVESRQLVISSAQE-DRNTQGGSLLTLALQTLVNQSP
Consensus   (697) IAGHDPSNIQILPCGFSIIPDGVESRQLVISSAQE DRNTQGGSLLTLALQTLVNQSP
```

Section 14

```
            (755) 755  760                770                             786
A.thaliana  (745) AAKLNMESVESVTNLVSVTLHNIKRSLQIEDC
B.napus-1   (719) AAKLNMESVESVTNLVSVTLHNIKRSLQIEDC
B.napus-2   (719) AAKLNMESVESVTNLVSVTLHNIKRCLQIEDC
B.oleracea  (719) AAKLNMESVESVTNLVSVTLHNIKRSLQIEDC
B.rapa      (719) AAKLNMESVESVTNLVSVTLHNIKRCLQIEDC
B.villosa   (717) AAKLNMESVESVTNLVSVTLHNIKRSLQIEDC
Consensus   (755) AAKLNMESVESVTNLVSVTLHNIKRSLQIEDC
```

GL2 orthologues and gene copies

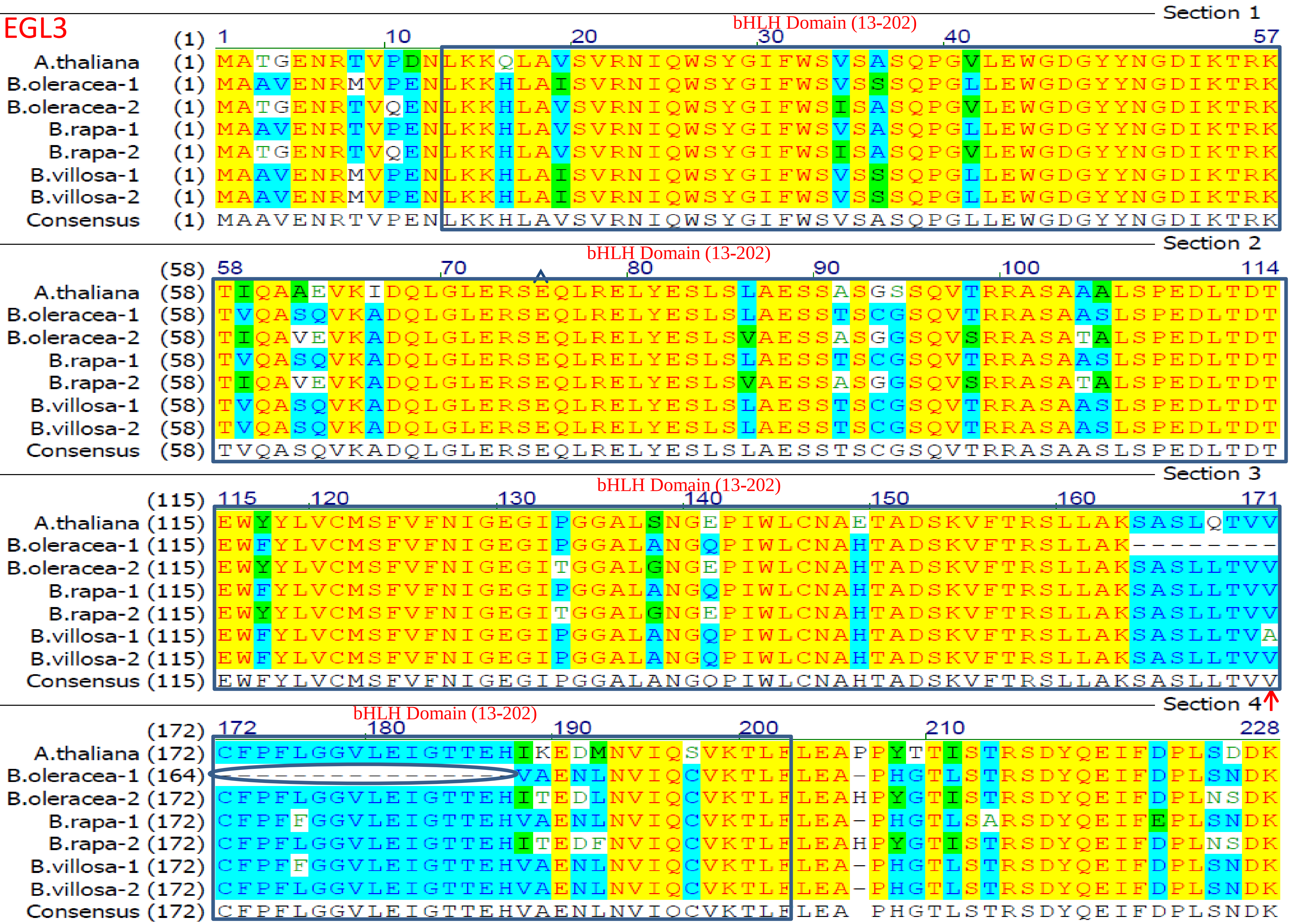

EGL3 orthologues and gene copies

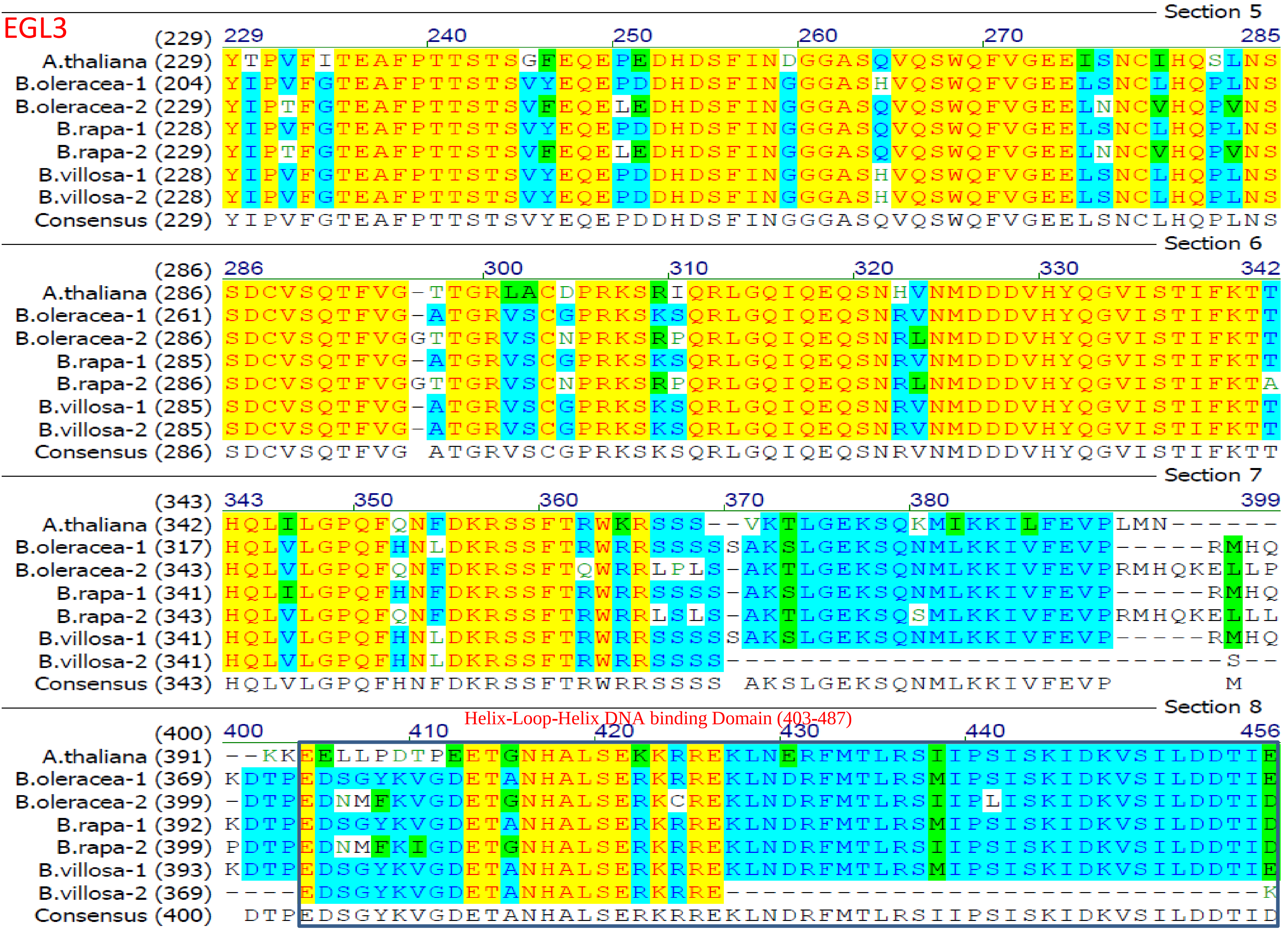

EGL3 orthologues and gene copies

EGL3

Helix-Loop-Helix DNA binding Domain (403-487)

Section 9

```
(457)            457          470            480            490            500            513
A.thaliana (446)  YLQDLQKRVQELESCRESADTETRITMMKRKKPDDEEERASANCMNSKRKGSDVNVG
B.oleracea-1 (426) YLQELQRRVQELESCRESIDTEMRMAMK-RKKPDGEDESASANCLNNKRKE--SDMG
B.oleracea-2 (455) YLQELQRRVQELESCREYTDTEMQMPMK-RKKPEDEDERASANCLNTKRKESDVNVG
B.rapa-1 (449)     YLQELQRRVQELESCRESTDTEMRMAMK-RKKPDGEDESASANCLNNKRKE--SDIG
B.rapa-2 (456)     YLQELQRRVQELESCREYTDTEMQMPMK-RKKPEDDDERASANCLNTKRKESDVNVG
B.villosa-1 (450)  YLQELQRRVQELESCRESIDTEMRMAMK-RKKPDGEDESASANCLNNKRKE--SDMG
B.villosa-2 (393)  LQTHLKITCLRLGMKPGTMPCPRGNAER-S---------------------------
Consensus (457)    YLQELQRRVQELESCRES DTEMRMAMK RKKPD EDE ASANCLN KRKE    VG
```

Section 10

```
(514)            514    520            530            540            550            560            570
A.thaliana (503)  EDEPAD-IGYAGLTDNLRISSLGNEVVIELRCAWREGILLEIMDVISDLNLDSHSVQ
B.oleracea-1 (480) EDEPAD-TGYAGLTDNLRIGSFGNEVVIELRCAWREGILLEIMDVISDLNLDSHSVQ
B.oleracea-2 (511) EDEPAD-TGYAGLTDNLRIGSFGNEVVIELRCAWREGILLEIMDVISHLNLDSHSVQ
B.rapa-1 (503)     EDEPAD-TGYAGLTDNLRIGSFGNEGVIELRCAWREGILLEIMDVISDLNLDSHSVQ
B.rapa-2 (512)     EDEPADDTGYAGLTDNLSIGSFGNEVVIELRCAWREGILLEIMDVISHLNLDSHSVQ
B.villosa-1 (504)  EDEPAD-TGYAGLTDNLRIGSFGNEVVIELRCAWREGIVLEIMDVISDLNLDSHSVQ
B.villosa-2 (422)  ---------------------------------------------------------
Consensus (514)    EDEPAD TGYAGLTDNLRIGSFGNEVVIELRCAWREGILLEIMDVISDLNLDSHSVQ
```

Section 11

```
(571)            571       580            590                 608
A.thaliana (559)  SSTGDGLLCLTVNCKHKGTKIATTGMIQEALQRVAWIC
B.oleracea-1 (536) SSTGDGLLCLTVNCKHKGTKIATTGMIQDALQRVAWIC
B.oleracea-2 (567) SSTGDGLLCLTVNCKHKGTNIATAGMIQEALQRVAWIC
B.rapa-1 (559)     SSTGDGLLCLTVNCKHKGTKIATTGMIQDALQRVAWIC
B.rapa-2 (569)     SSTGDGLLCLTVNCKHKGTNIATAGMIQEALQRVAWIC
B.villosa-1 (560)  SSTGDGLLCLTVNCKHKGTKIATTGMIQDALQRVAWIC
B.villosa-2 (422)  --------------------------------------
Consensus (571)    SSTGDGLLCLTVNCKHKGTKIATTGMIQDALQRVAWIC
```

EGL3 orthologues and gene copies

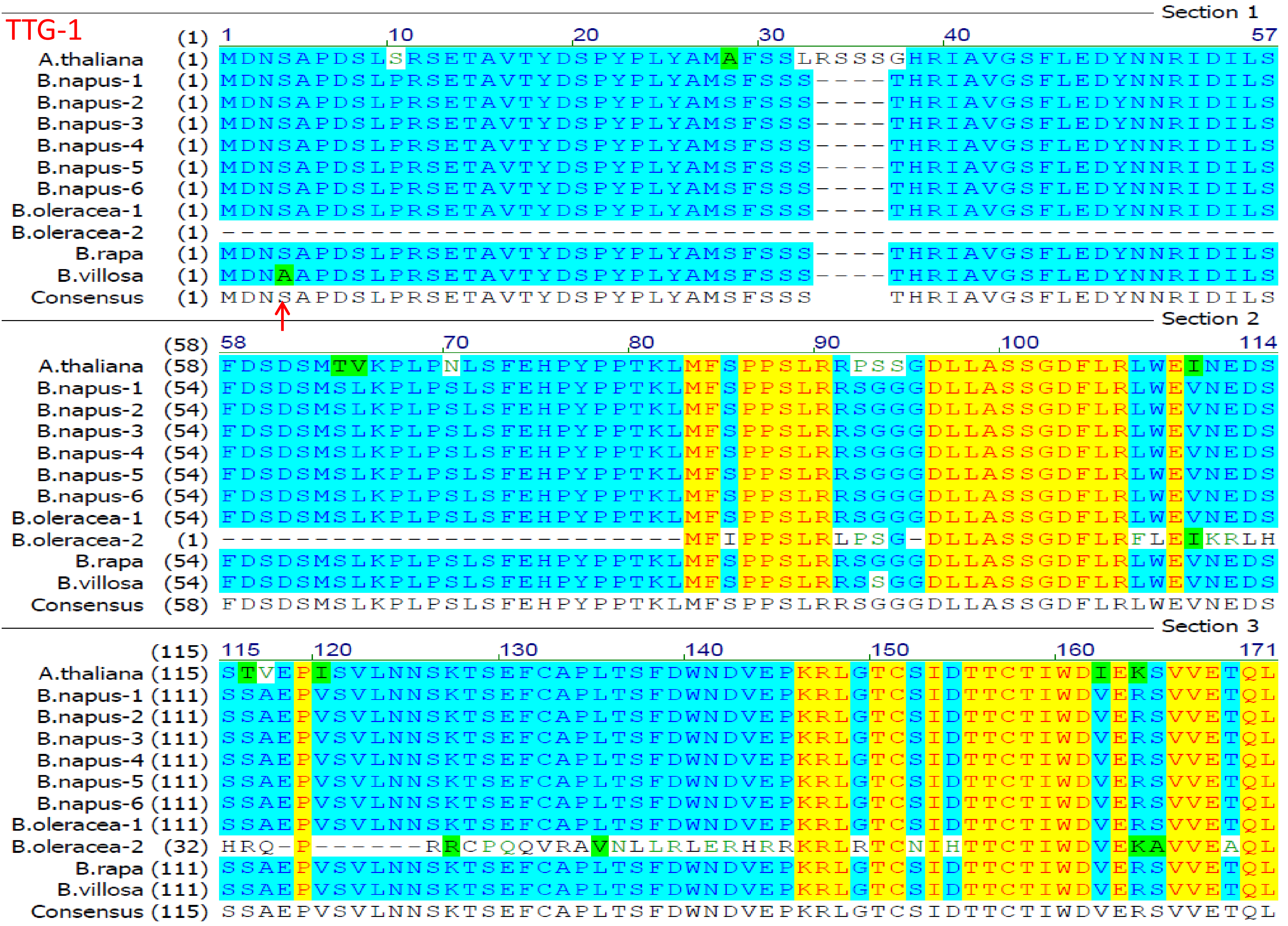

TTG1 orthologues and gene copies

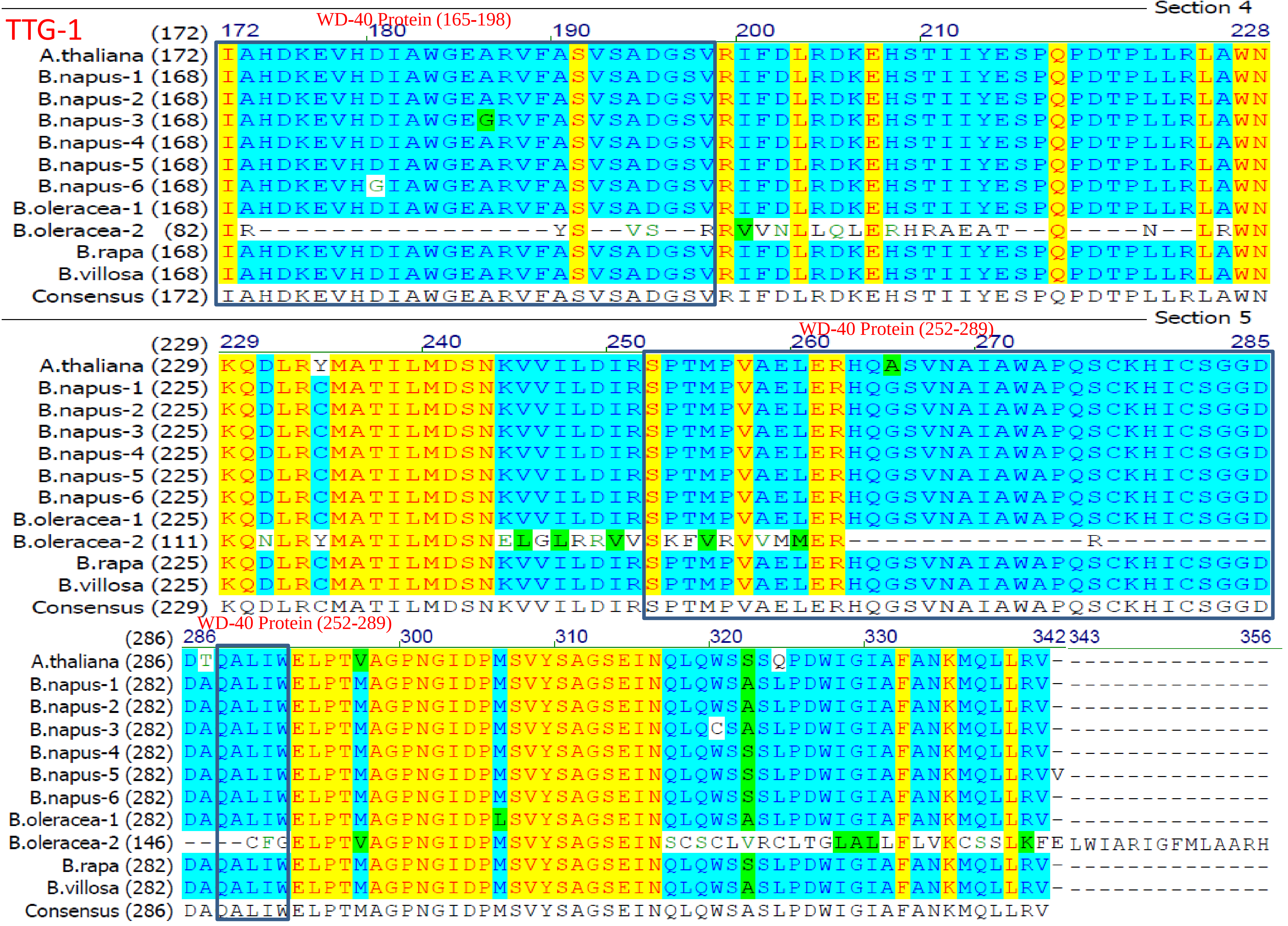

TTG1 orthologues and gene copies

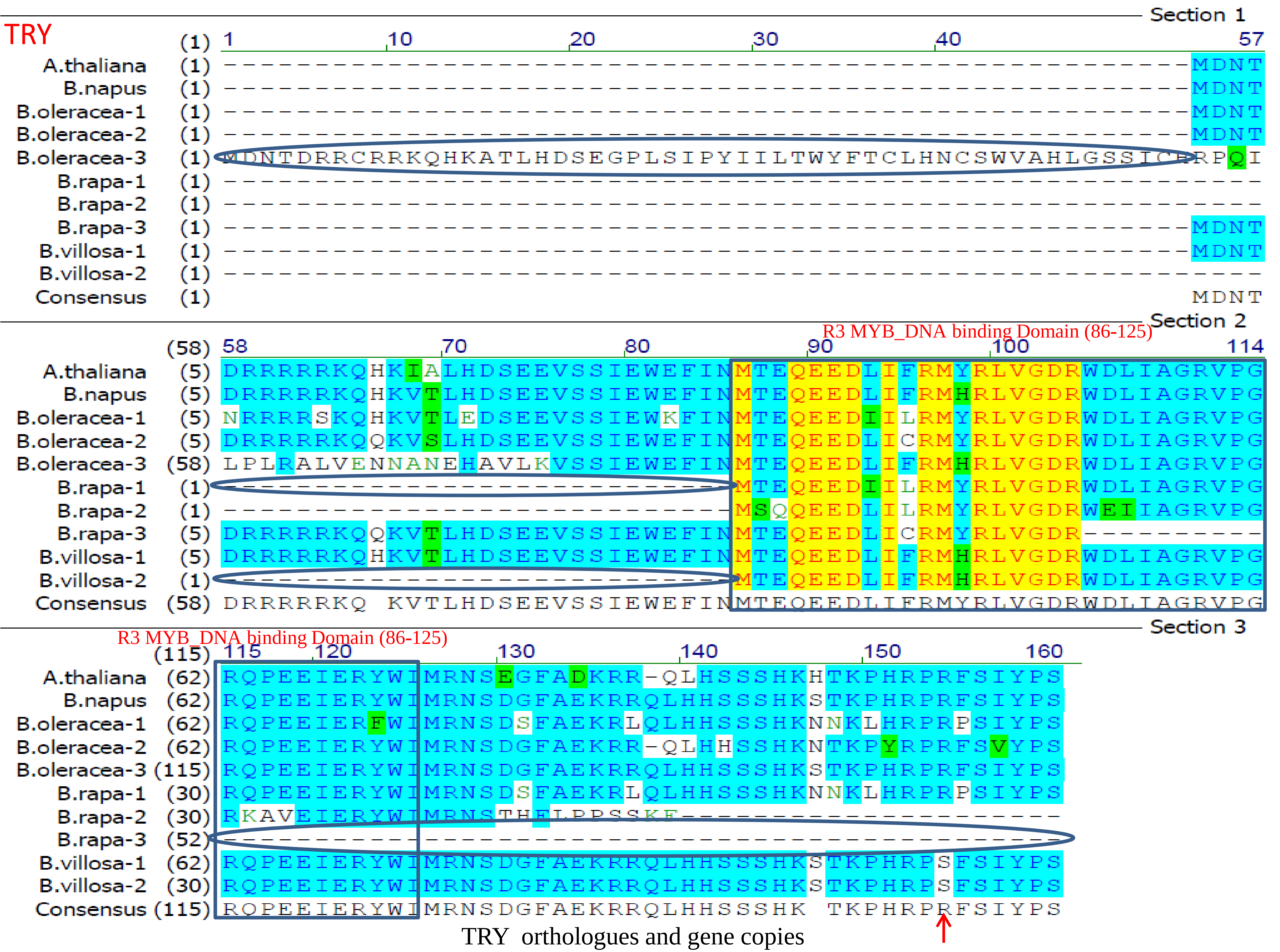

TRY orthologues and gene copies
