# Supplementary figures and images for: Comparison of Five Major Trichome Regulatory Genes in Brassica villosa with Orthologues within the Brassicaceae
Source: PLoS One. 2014 Apr 22;9(4):e95877. doi: 10.1371/journal.pone.0095877 (PMC3995807; doi:10.1371/journal.pone.0095877)

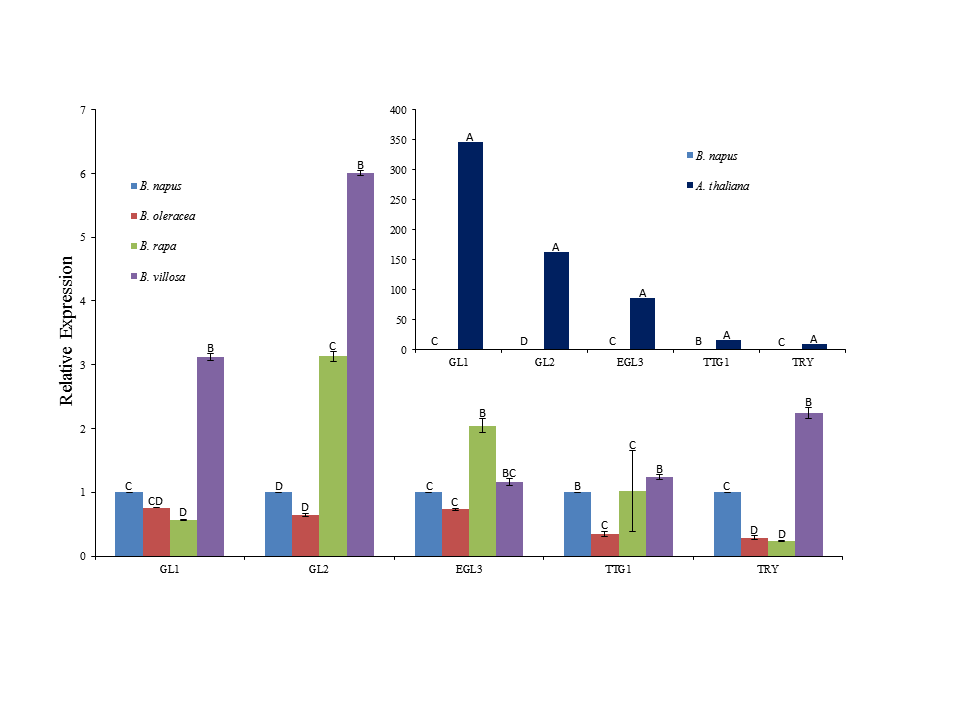

Supplement: Figure S2 — Relative expression of four trichome regulatory genes and one negative regulatory gene in leaves of hairy Brassica villosa, three other Brassica species, and A. thaliana. Main panel shows expressed transcripts from four Brassica species. Insert panel shows A. thaliana orthologues which are much more highly expressed compared with the Brassica species. Expression (Q-PCR) in both panels is relative to glabrous B. napus cv. Westar (set at 1). Different letters in both panels indicate significant differences of the means (± standard error) at p≤0.05 in an LSD test (SAS, 2008). (TIF) [file pone.0095877.s002.tif]

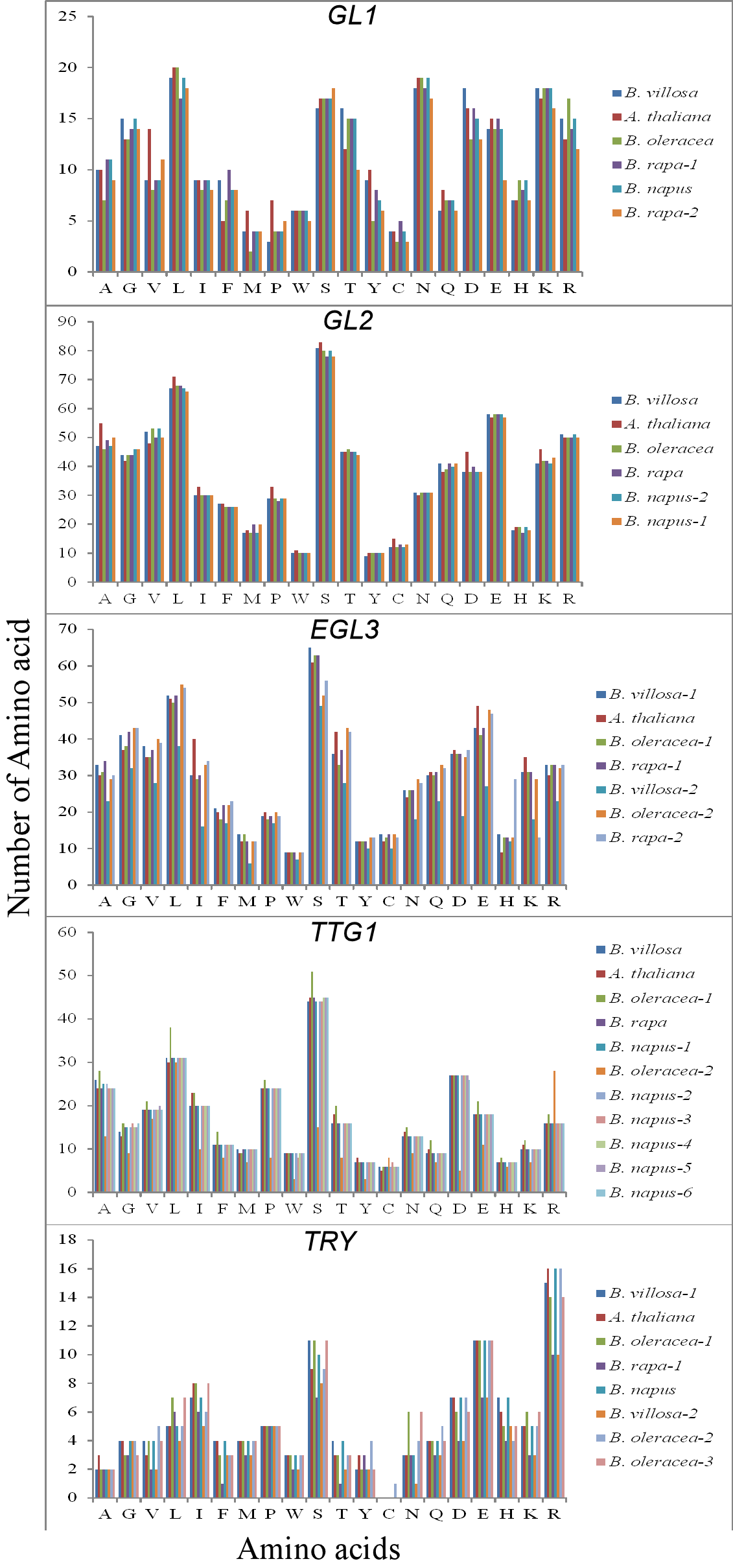

Supplement: Figure S3 — Amino acid profiles for five major trichome regulatory sequences from Brassica villosa, three other Brassica species, and A. thaliana. (TIF) [file pone.0095877.s003.tif]
