# Supplementary material for: Comparison of Five Major Trichome Regulatory Genes in Brassica villosa with Orthologues within the Brassicaceae
Source: PLoS One. 2014 Apr 22;9(4):e95877. doi: 10.1371/journal.pone.0095877 (PMC3995807; doi:10.1371/journal.pone.0095877)
Supplement: Table S1 — *Ka/Ks values from pairwise comparisons of all trichome regulatory gene orthologues and homologues within four Brassica species and Arabidopsis. (DOCX) [file pone.0095877.s004.docx]

Table S1: **Ka*/*Ks* values from pairwise comparisons of all trichome regulatory gene orthologues and homologues within four *Brassica* species and Arabidopsis.

| Pairwise comparison | | Ka | Ks | Ka/Ks | Ka/Ks alignment region  Protein % Nucleotide % | |
| --- | --- | --- | --- | --- | --- | --- |
| GL1 |  |  |  |  |  |  |
| *B. oleracea* | *B. napus* | 0.0405 | 0.0502 | 0.80675 | 93.87 | 96.38 |
| *B. oleracea* | *B. villosa* | 0.0516 | 0.066 | 0.78194 | 91.98 | 95.44 |
| *B. oleracea* | *B. rapa-*1 | 0.0644 | 0.0849 | 0.7582 | 89.62 | 94.18 |
| *B. oleracea* | *B. rapa-*2 | 0.1311 | 0.5426 | 0.24162 | 77.9 | 82.5 |
| *B. oleracea* | *A. thaliana* | 0.1811 | 0.7137 | 0.25377 | 71.43 | 79.05 |
| *B. napus* | *B. villosa* | 0.0214 | 0.0114 | 1.8697 | 96.44 | 98.52 |
| *B. napus* | *B. rapa-*1 | 0.0333 | 0.0318 | 1.04692 | 94.22 | 97.04 |
| *B. napus* | *B. rapa-*2 | 0.0968 | 0.4343 | 0.22285 | 82.29 | 86.28 |
| *B. napus* | *A. thaliana* | 0.1499 | 0.6364 | 0.23561 | 74.89 | 81.02 |
| *B. villosa* | *B. rapa-*1 | 0.0373 | 0.036 | 1.03554 | 93.33 | 96.74 |
| *B. villosa* | *B. rapa-*2 | 0.094 | 0.4384 | 0.21432 | 82.29 | 86.46 |
| *B. villosa* | *A. thaliana* | 0.1517 | 0.6246 | 0.24283 | 74.89 | 81.02 |
| *B. rapa-*1 | *B. rapa-*2 | 0.1036 | 0.4413 | 0.23464 | 80.73 | 85.76 |
| *B. rapa*-1 | *A. thaliana* | 0.1353 | 0.594 | 0.22787 | 77.13 | 82.36 |
| *B. rapa*-2 | *A. thaliana* | 0.1991 | 0.6146 | 0.32389 | 67.54 | 78.53 |
| GL2 |  |  |  |  |  |  |
| *B. oleracea* | *B. napus-*2 | 0.0018 | 0.0307 | 0.05984 | 99.6 | 99.11 |
| *B. oleracea* | *B. villosa* | 0.0044 | 0.0326 | 0.13507 | 99.06 | 98.84 |
| *B. oleracea* | *B. rapa* | 0.0098 | 0.1444 | 0.0679 | 97.59 | 96.07 |
| *B. oleracea* | *B. napus*-1 | 0.0073 | 0.1255 | 0.05804 | 98.26 | 96.74 |
| *B. oleracea* | *A. thaliana* | 0.0346 | 0.4692 | 0.07376 | 92.71 | 89.07 |
| *B. napus*-2 | *B. villosa* | 0.0024 | 0.0278 | 0.08539 | 99.47 | 99.2 |
| *B. napus*-2 | *B. rapa* | 0.008 | 0.1265 | 0.06302 | 97.99 | 96.57 |
| *B. napus*-2 | *B. napus*-1 | 0.0055 | 0.1082 | 0.05047 | 98.66 | 97.24 |
| *B. napus*-2 | *A. thaliana* | 0.0328 | 0.4698 | 0.06972 | 93.12 | 89.16 |
| *B. villosa* | *B. rapa* | 0.0079 | 0.1466 | 0.05392 | 97.99 | 96.21 |
| *B. villosa* | *B. napus*-1 | 0.0054 | 0.1312 | 0.04137 | 98.66 | 96.79 |
| *B. villosa* | *A. thaliana* | 0.0327 | 0.4821 | 0.06774 | 92.98 | 89.02 |
| *B. rapa* | *B. napus-*1 | 0.0038 | 0.0268 | 0.14169 | 99.07 | 98.98 |
| *B. rapa* | *A. thaliana* | 0.0374 | 0.4779 | 0.07822 | 91.91 | 88.68 |
| *B. napus*-1 | *A. thaliana* | 0.0347 | 0.4702 | 0.07375 | 92.58 | 89.02 |
| EGL3 |  |  |  |  |  |  |
| *B. villosa* -1 | *B. oleracea* -1 | 0 | 0 | 0.51373 | 99.83 | 99.88 |
| *B. villosa* -1 | *B. rapa*-1 | 0.0098 | 0.0317 | 0.30739 | 97.65 | 98.43 |
| *B. villosa* -1 | *B. villosa* -2 | 0.0448 | 0.0522 | 0.85783 | 94.06 | 95.72 |
| *B. villosa* -1 | *B. oleracea*-2 | 0.0702 | 0.234 | 0.29983 | 87.08 | 89.82 |
| *B. villosa* -1 | *B. rapa*-2 | 0.0688 | 0.216 | 0.31839 | 86.74 | 89.88 |
| *B. villosa* -1 | *A. thaliana* | 0.0809 | 0.3999 | 0.20224 | 85.18 | 86.43 |
| *B. oleracea-*1 | *B. rapa*-1 | 0.0098 | 0.0317 | 0.30739 | 97.9 | 98.6 |
| *B. oleracea* -1 | *B. villosa* -2 | 0.0448 | 0.0522 | 0.85783 | 94.21 | 95.63 |
| *B. oleracea* -1 | *B. oleracea*-2 | 0.0702 | 0.234 | 0.29983 | 87.06 | 89.69 |
| *B. oleracea* -1 | *B. rapa*-2 | 0.0688 | 0.216 | 0.31839 | 86.71 | 89.8 |
| *B. oleracea* -1 | *A. thaliana* | 0.0809 | 0.3999 | 0.20224 | 85.26 | 86.26 |
| *B. rapa*-1 | *B. villosa* -2 | 0.0545 | 0.0918 | 0.59326 | 92.4 | 94.14 |
| *B. rapa*-1 | *B. oleracea*-2 | 0.0677 | 0.259 | 0.26145 | 88.07 | 89.75 |
| *B. rapa*-1 | *B. rapa*-2 | 0.0662 | 0.2324 | 0.28483 | 87.73 | 89.92 |
| *B. rapa*-1 | *A. thaliana* | 0.0761 | 0.4109 | 0.18522 | 86.01 | 86.63 |
| *B. villosa* -2 | *B. oleracea*-2 | 0.1176 | 0.2995 | 0.39284 | 81.71 | 85.99 |
| *B. villosa* -2 | *B. rapa*-2 | 0.1163 | 0.2789 | 0.41702 | 81.71 | 86.22 |
| *B. villosa* -2 | *A. thaliana* | 0.1195 | 0.4151 | 0.28778 | 81.31 | 84.55 |
| *B. oleracea*-2 | *B. rapa*-2 | 0.0062 | 0.0278 | 0.22188 | 98.34 | 98.73 |
| *B. oleracea*-2 | *A. thaliana* | 0.0759 | 0.3881 | 0.19552 | 87.21 | 87.49 |
| *B. rapa*-2 | *A. thaliana* | 0.0741 | 0.3673 | 0.20161 | 87.39 | 87.68 |
| TTG1 |  |  |  |  |  |  |
| *B. napus*-4 | *B. napus-*5 | 0 | 0 | 0.39257 | 100 | 100 |
| *B. napus*-4 | *B. rapa* | 0 | 0.0226 | 0.001 | 100 | 99.31 |
| *B. napus*-4 | *B. napus-*6 | 0 | 0 | 0.001 | 99.7 | 99.9 |
| *B. napus*-4 | *B. oleracea-*1 | 0.0041 | 0.1343 | 0.03038 | 99.41 | 97.73 |
| *B. napus*-4 | *B. napus*-1 | 0.002 | 0.2245 | 0.00905 | 99.7 | 97.13 |
| *B. napus*-4 | *B. napus*-2 | 0.002 | 0.2245 | 0.00905 | 99.7 | 97.13 |
| *B. napus*-4 | *B. napus*-3 | 0.0041 | 0.2136 | 0.01899 | 99.11 | 97.13 |
| *B. napus*-4 | *B. villosa* | 0.0041 | 0.1421 | 0.0289 | 99.11 | 97.63 |
| *B. napus*-4 | *A. thaliana* | 0.026 | 2.4953 | 0.01043 | 94.07 | 81.31 |
| *B. napus*-4 | *B. oleracea-*2 | 0.5091 | 1.4503 | 0.35101 | 48.22 | 58.88 |
| *B. napus*-5 | *B. rapa* | 0 | 0.0225 | 0.001 | 100 | 99.31 |
| *B. napus*-5 | *B. napus*-6 | 0 | 0 | 0.001 | 99.7 | 99.9 |
| *B. napus*-5 | *B. oleracea-*1 | 0.0041 | 0.1343 | 0.03038 | 99.41 | 97.73 |
| *B. napus-*5 | *B. napus*-1 | 0.002 | 0.2245 | 0.00905 | 99.7 | 97.13 |
| *B. napus*-5 | *B. napus-*2 | 0.002 | 0.2245 | 0.00905 | 99.7 | 97.13 |
| *B. napus*-5 | *B. napus*-3 | 0.0041 | 0.2136 | 0.01899 | 99.11 | 97.13 |
| *B. napus*-5 | *B. villosa* | 0.0041 | 0.1421 | 0.0289 | 99.11 | 97.63 |
| *B. napus*-5 | *A. thaliana* | 0.026 | 2.4954 | 0.01043 | 94.07 | 81.31 |
| *B. napus*-5 | *B. oleracea-*2 | 0.5091 | 1.4503 | 0.35101 | 48.22 | 58.88 |
| *B. rapa* | *B. napus*-6 | 0 | 0.0226 | 0.001 | 99.7 | 99.21 |
| *B. rapa* | *B. oleracea-*1 | 0.0041 | 0.1631 | 0.02488 | 99.41 | 97.63 |
| *B. rapa* | *B. napus*-1 | 0.002 | 0.2588 | 0.00781 | 99.7 | 97.23 |
| *B. rapa* | *B. napus*-2 | 0.002 | 0.2588 | 0.00781 | 99.7 | 97.23 |
| *B. rapa* | *B. napus*-3 | 0.004 | 0.2471 | 0.01634 | 99.11 | 97.03 |
| *B. rapa* | *B. villosa* | 0.0041 | 0.1715 | 0.02379 | 99.11 | 97.53 |
| *B. rapa* | *A. thaliana* | 0.0261 | 2.4275 | 0.01076 | 94.07 | 81.21 |
| *B. rapa* | *B. oleracea-*2 | 0.5107 | 1.4021 | 0.36425 | 48.22 | 59.05 |
| *B. napus*-6 | *B. oleracea-*1 | 0.0041 | 0.1343 | 0.03038 | 99.11 | 97.63 |
| *B. napus*-6 | *B. napus*-1 | 0.002 | 0.2245 | 0.00905 | 99.41 | 97.03 |
| *B. napus*-6 | *B. napus*-2 | 0.002 | 0.2245 | 0.00905 | 99.41 | 97.03 |
| *B. napus*-6 | *B. napus*-3 | 0.0041 | 0.2136 | 0.01899 | 98.81 | 97.03 |
| *B. napus*-6 | *B. villosa* | 0.0041 | 0.1421 | 0.0289 | 98.81 | 97.53 |
| *B. napus*-6 | *A. thaliana* | 0.026 | 2.4953 | 0.01043 | 93.77 | 81.21 |
| *B. napus*-6 | *B. oleracea-*2 | 0.5091 | 1.4503 | 0.35101 | 48.22 | 58.88 |
| *B. oleracea-*1 | *B. napus*-1 | 0.002 | 0.1125 | 0.01808 | 99.7 | 98.52 |
| *B. oleracea-*1 | *B. napus*-2 | 0.002 | 0.1125 | 0.01808 | 99.7 | 98.52 |
| *B. oleracea-*1 | *B. napus*-3 | 0.0041 | 0.1031 | 0.03929 | 99.11 | 98.52 |
| *B. oleracea-*1 | *B. villosa* | 0.0042 | 0.0405 | 0.10304 | 99.11 | 98.62 |
| *B. oleracea-*1 | *A. thaliana* | 0.0308 | 1.9519 | 0.01579 | 93.47 | 80.91 |
| *B. oleracea-*1 | *B. oleracea-*2 | 0.5062 | 1.8151 | 0.27888 | 47.72 | 58.04 |
| *B. napus*-1 | *B. napus*-2 | 0 | 0 | 0.01471 | 100 | 100 |
| *B. napus*-1 | *B. napus*-3 | 0.0021 | 0.0102 | 0.20203 | 99.41 | 99.6 |
| *B. napus*-1 | *B. villosa* | 0.0021 | 0.0837 | 0.02466 | 99.41 | 98.62 |
| *B. napus*-1 | *A. thaliana* | 0.0284 | 2.3381 | 0.01215 | 93.77 | 80.61 |
| *B. napus*-1 | *B. oleracea-*2 | 0.4681 | 63.1562 | 0.00741 | 48.22 | 57.53 |
| *B. napus*-2 | *B. napus*-3 | 0.0021 | 0.0102 | 0.20203 | 99.41 | 99.6 |
| *B. napus*-2 | *B. villosa* | 0.0021 | 0.0837 | 0.02466 | 99.41 | 98.62 |
| *B. napus*-2 | *A. thaliana* | 0.0284 | 2.3381 | 0.01215 | 93.77 | 80.61 |
| *B. napus*-2 | *B. oleracea-*2 | 0.4682 | 62.055 | 0.00754 | 48.22 | 57.53 |
| *B. napus*-3 | *B. villosa* | 0.0041 | 0.075 | 0.05463 | 98.81 | 98.42 |
| *B. napus*-3 | *A. thaliana* | 0.0306 | 2.2175 | 0.01382 | 93.18 | 80.61 |
| *B. napus*-3 | *B. oleracea-*2 | 0.465 | 49.3465 | 0.00942 | 48.73 | 57.53 |
| *B. villosa* | *A. thaliana* | 0.0284 | 2.1606 | 0.01314 | 93.77 | 81.01 |
| *B. villosa* | *B. oleracea-*2 | 0.4984 | 1.8701 | 0.26653 | 48.73 | 58.21 |
| *A. thaliana* | *B. oleracea-*2 | 0.4802 | 2.8048 | 0.17122 | 50.76 | 56.68 |
| TRY |  |  |  |  |  |  |
| *B. villosa*-2 | *B. oleracea*-1 | 0.0054 | 0.0871 | 0.06236 | 98.67 | 98.22 |
| *B. villosa-*2 | *B. villosa*-1 | 0 | 0 | 0.001 | 100 | 100 |
| *B. villosa*-2 | *B. napus* | 0.0056 | 0.0243 | 0.23097 | 98.67 | 99.11 |
| *B. villosa*-2 | *A. thaliana* | 0.0355 | 0.5663 | 0.06273 | 93.24 | 88.74 |
| *B. villosa*-2 | *B. oleracea*-2 | 0.047 | 0.3046 | 0.15423 | 90.54 | 91.44 |
| *B. villosa*-2 | *B. oleracea*-3 | 0.0735 | 0.3938 | 0.18658 | 85.33 | 88.44 |
| *B. villosa*-2 | *B. rapa* | 0.0682 | 0.3552 | 0.192 | 86.67 | 89.33 |
| *B. oleracea*-1 | *B. villosa*-1 | 0.0054 | 0.0871 | 0.06236 | 96.26 | 97.82 |
| *B. oleracea*-1 | *B. napus* | 0.0001 | 0.0559 | 0.001 | 97.2 | 98.44 |
| *B. oleracea*-1 | *A. thaliana* | 0.0292 | 0.6192 | 0.04721 | 92.45 | 89.62 |
| *B. oleracea*-1 | *B. oleracea*-2 | 0.0408 | 0.2717 | 0.15 | 89.62 | 92.14 |
| *B. oleracea*-1 | *B. oleracea*-3 | 0.0667 | 0.3739 | 0.1783 | 84.11 | 88.47 |
| *B. oleracea*-1 | *B. rapa* | 0.0604 | 0.4097 | 0.14752 | 88 | 89.33 |
| *B. villosa*-1 | *B. napus* | 0.0056 | 0.0243 | 0.23097 | 99.07 | 99.38 |
| *B. villosa*-1 | *A. thaliana* | 0.0355 | 0.5663 | 0.06273 | 93.4 | 90.57 |
| *B. villosa*-1 | *B. oleracea*-2 | 0.047 | 0.3046 | 0.15423 | 91.51 | 92.45 |
| *B. villosa*-1 | *B. oleracea*-3 | 0.0735 | 0.3938 | 0.18658 | 85.98 | 88.79 |
| *B. villosa*-1 | *B. rapa* | 0.0682 | 0.3552 | 0.192 | 86.67 | 89.33 |
| *B. napus* | *A. thaliana* | 0.0295 | 0.5258 | 0.05607 | 94.34 | 91.19 |
| *B. napus* | *B. oleracea-*2 | 0.0408 | 0.2737 | 0.14897 | 92.45 | 93.08 |
| *B. napus* | *B. oleracea*-3 | 0.0668 | 0.3623 | 0.18434 | 86.92 | 89.41 |
| *B. napus* | *B. rapa* | 0.0614 | 0.3249 | 0.18914 | 88 | 90.22 |
| *A. thaliana* | *B. oleracea*-2 | 0.0481 | 0.7645 | 0.06286 | 90.57 | 88.68 |
| *A. thaliana* | *B. oleracea*-3 | 0.0736 | 0.9979 | 0.07374 | 83.96 | 83.65 |
| *A. thaliana* | *B. rapa* | 0.0677 | 0.9485 | 0.07143 | 86.49 | 83.33 |
| *B. oleracea*-1 | *B. oleracea*-3 | 0.0851 | 0.7387 | 0.11523 | 83.96 | 85.53 |
| *B. oleracea*-2 | *B. rapa* | 0.0792 | 0.6986 | 0.11341 | 86.49 | 85.14 |
| *B. oleracea*-3 | *B. rapa* | 0.0055 | 0.0908 | 0.06005 | 98.67 | 98.22 |

**Ka*/*K_S_* (Yang Z, 1997): *Ka*, non-synonymous nucleotide substitution. *Ks*, synonymous nucleotide substitution value. *B. rapa*-2 (BrTRY-2) and *B. rapa*-3 (BrTRY-3) amino acid sequences were too short to be included. NA, *B. napus* sequence not available.
